# Different Correlation Patterns Between Circulating Amino Acids and Body Temperature in Fibromyalgia Syndrome: A Cross-Sectional Study

**DOI:** 10.3390/ijms252413517

**Published:** 2024-12-17

**Authors:** Antonio Casas-Barragán, Francisco Molina, Rosa María Tapia-Haro, José Manuel Martínez-Martos, María Jesús Ramírez-Expósito, Alma Rus, María Correa-Rodríguez, María Encarnación Aguilar-Ferrándiz

**Affiliations:** 1 Department of Physical Therapy, Faculty of Health Sciences, University of Granada (UGR), 18071 Granada, Spain; antoniocb@ugr.es (A.C.-B.); fjmolina@ugr.es (F.M.); rtapia@ugr.es (R.M.T.-H.); e_aguilar@ugr.es (M.E.A.-F.); 2 Instituto de Investigación Biosanitaria ibs.GRANADA, 18012 Granada, Spain; mrus@ugr.es; 3 Department of Health Sciences, University of Jaén (UJA), 23071 Jaén, Spain; jmmartos@ujaen.es (J.M.M.-M.); mramirez@ujaen.es (M.J.R.-E.); 4 Department of Cell Biology, University of Granada (UGR), 18071 Granada, Spain; 5 Department of Nursing, Faculty of Health Sciences, University of Granada (UGR), 18071 Granada, Spain

**Keywords:** fibromyalgia, thermography, core body temperature, amino acids, thermoregulation

## Abstract

The aim of this study was to analyze the association between circulating amino acids and central and peripheral body temperature in subjects with and without fibromyalgia syndrome (FMS). A total of 47 patients with FMS and 59 healthy subjects were included in the study. The concentration of amino acids was determined in serum samples using a fluorimeter coupled with a high-performance liquid chromatography system. An infrared thermography camera was used to estimate peripheral hand temperatures. The core temperature of the body was estimated using an infrared thermometer, which was applied to the axillary and tympanic areas. Correlations between several thermographic variables of the hands and tryptophan, methionine, 3-methylhistidine, histidine, glutamic acid, and tyrosine were identified exclusively within the FMS group. In contrast, correlations between aminoadipic acid and serine and thermographic variables were observed only in the healthy control group. The concentrations of asparagine and lysine correlated with thermographic variables in both groups. The essential amino acid leucine was found to correlate with axillary temperature in FMS patients. However, it should be noted that the observed associations between aminoadipic acid and tryptophan blood concentrations and axillary temperature were limited to the control group. Several correlations were identified between circulating amino acids and different body temperatures in both healthy controls and patients with FMS. However, the correlation pattern differs significantly between FMS patients and healthy controls. These findings suggest the possibility of a change in the function of several amino acids in the thermoregulatory process in patients with FMS.

## 1. Introduction

Fibromyalgia syndrome (FMS) is characterized by the presence of chronic musculoskeletal pain in combination with several symptoms, including hyperalgesia, anxiety, sleep disturbances, and chronic fatigue [1]. The global prevalence of FMS is estimated to be 2–4%, with a pronounced female predominance (4.2% in females versus 0.2% in males) [2]. Within the clinical complexity of FMS, one of the most curious symptoms is related to the central processing of thermal sensation. A review of the scientific literature reveals that patients with FMS exhibit impairments in thermal sensation perception [3]. Furthermore, fluctuations in ambient temperature have been observed to exacerbate the primary symptoms experienced by individuals with FMS, including pain and disability [4]. This preliminary finding indicates a potential correlation between thermoregulation and FMS symptoms. Peripheral thermal perception is the initial stage of a complex process known as thermoregulation [5].

There are currently no clinical measures or specific biomarkers to accurately diagnose FMS. A diagnosis of FMS can be made if the patient meets the following 2016 American College of Rheumatology (ACR) criteria: (1) generalized pain in at least four of the following five regions: right upper body region, left upper body region, right lower body region, left lower body region, and axial region; (2) symptoms present for at least 3 months; (3) high scores on the Widespread Pain Index (WPI) or the Symptom Severity Scale (SSS) [6]. Several treatments have been demonstrated to be effective in alleviating the symptoms and improving quality of life in patients with FMS. FMS treatment is multidisciplinary, primarily through pharmacotherapy, and includes other non-pharmacological therapies such as physical exercise, meditation (e.g., Qi-gong, yoga, or Tai Chi), cognitive behavioral therapy, mindfulness, transcranial magnetic stimulation, or physiotherapy, among others [7].

Thermoregulation is the capacity of a homeothermic organism to maintain a constant body core temperature despite external temperature fluctuations. As the external temperature rises, the body eliminates the excess of internal heat, augmenting the vasodilation of specific parts of the body that act as radiators. Conversely, the occurrence of an external cold situation results in a powerful vasoconstriction of these body parts, which prevents the loss of corporal heat. These regions of the body, called glabrous skin, correspond with non-hairy skin and are predominantly observed in the feet and hands [8]. They have a specific network of blood vessels dedicated to the thermoregulatory process. In order to analyze the manner in which these parts of the body preserve or emit body heat, thermography techniques are typically employed [9]. Several factors influence the tone of specific blood vessels located in glabrous skin, including glucose [10] and circulating amino acids [11], which modulate the thermoregulatory process using the control of brown adipose tissue metabolism [12] and vascular tone [11]. Additionally, some works have proposed that FMS patients have different concentrations of several amino acids compared to age-matched healthy controls [13] or other rheumatic diseases [14]. In addition, recent studies have suggested that defective deactivation of RNAse L due to an overexpression of interferon may trigger the fragmentation of some transfer RNAs in immune cells obtained from FMS patients [15]. This degradation could modify the bioavailability of some amino acids and alter protein synthesis and function [16].

In summary, the scientific literature suggests a link between the inadequate thermoregulation observed in several diseases and a specific amino acids profile in these patients [12]. Therefore, the present study analyzed the relationship between the concentration of several circulating amino acids and peripheral (hand thermography) or central temperature in patients with FMS compared to healthy controls.

## 2. Results

### 2.1. Demographic Data, Peripheral Skin Temperature, Central Temperature, and Amino Acid Concentrations

**Figure 1:**
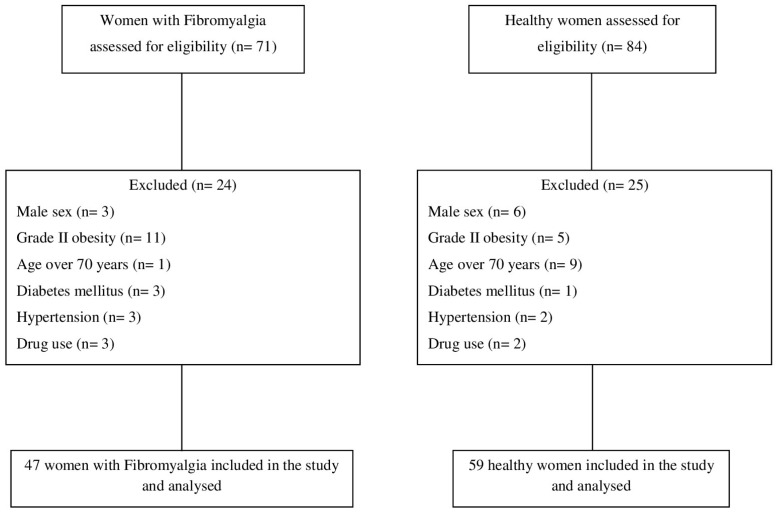
Flow diagram of the patient screening process for study enrollment.

A flow diagram illustrating the participant selection process throughout the study is shown in Figure 1. The demographic data, peripheral skin temperature of the hands, central temperature, and amino acid concentrations are presented in Table 1. The median ages for the FMS women and healthy women were 55 and 52 years, respectively. The women diagnosed with FMS exhibited a high level of disease severity (mean Revised Fibromyalgia Impact Questionnaire (FIQ-R) score ± standard deviation (SD) = 72.02 ± 16.93). In addition, our population of women with FMS presented a mean visual analog scale (VAS) score ± SD of 73.62 ± 18.58. The mean dorsal and palmar temperatures of both hands of women with FMS were significantly higher than those of healthy women (*p* < 0.05). Women with FMS also had significantly higher tympanic temperatures than controls (*p* = 0.023). However, there were no significant differences in age, height, weight, BMI, age of menarche, age of menopause, axillary temperature, and serine, citrulline, carnosine, GABA, or lysine concentrations between the study groups.

### 2.2. Correlations Between Amino Acids and Hand Temperature

The overall analysis of amino acid groups shows only a statistically significant correlation between EAAs and the temperature of the dorsum of the dominant hand in patients diagnosed with FMS. For this reason, we analyzed the correlation for each specific amino acid with hand temperature. The essential amino acid tryptophan (Trp) exhibited a total of fifteen positive correlations with thermographic variables of the palm and dorsum of the hands, exclusively in patients diagnosed with FMS (Tables S1 and S2). Similarly, the essential amino acids methionine (Met) and lysine (Lys) have eleven and one significant positive correlations, respectively, with several hand temperatures of FMS patients (Tables S1 and S2). The 3-methylhistidine (3-MH) concentration correlates with nine hand thermographic variables only in FMS patients (Tables S1 and S2). Finally, the amino acid histidine (His) was significantly associated with the average temperature of the dorsal center of the dominant hand in the FMS group (Table S2). On the other hand, the non-essential amino acids, glutamic acid (Glu), and asparagine (Asn) have fourteen and two positive correlations with several thermographic variables in FMS patients (Tables S1 and S2). Additionally, the non-essential amino acid tyrosine (Tyr) has three positive correlations with the thermographic variables, exclusively in individuals diagnosed with FMS. No statistically significant correlations were identified between thermographic variables of FMS patients and the concentrations of aminoadipic acid, arginine, aspartic acid, citrulline, carnosine, phenylalanine, GABA, glycine, glutamine, isoleucine, leucine, ornithine, serine, taurine, threonine, or valine.

In the control group, only Asn concentrations exhibited statistically significant positive correlations with several thermographic variables of the hands (Tables S1 and S2). It is noteworthy that the concentrations of aminoadipic acid, lysine, and serine exhibited inverse correlations with several hand temperatures exclusively within the healthy control group (Tables S1 and S2).

### 2.3. Correlations Between Amino Acids and Core Temperature

Only leucine concentrations showed significant positive associations with tympanic and axillary temperatures in FMS patients (Table S3). No further correlations were found between body core temperature and other amino acids analyzed in patients diagnosed with FMS.

In the control group, the essential amino acid tryptophan exhibited a positive correlation with both axillary temperature and the difference between tympanic and axillary temperatures (Table S3). However, a negative correlation was observed between the aminoadipic acid concentration and axillary temperature (Table S3). No significant interactions were found between the core body temperatures of healthy women and the rest of the amino acids analyzed (Table S3).

### 2.4. Final Multiple Regression Model of Predictive Factors Associated with Peripheral and Central Temperature in Women with FMS and Healthy Women

**Figure 2:**
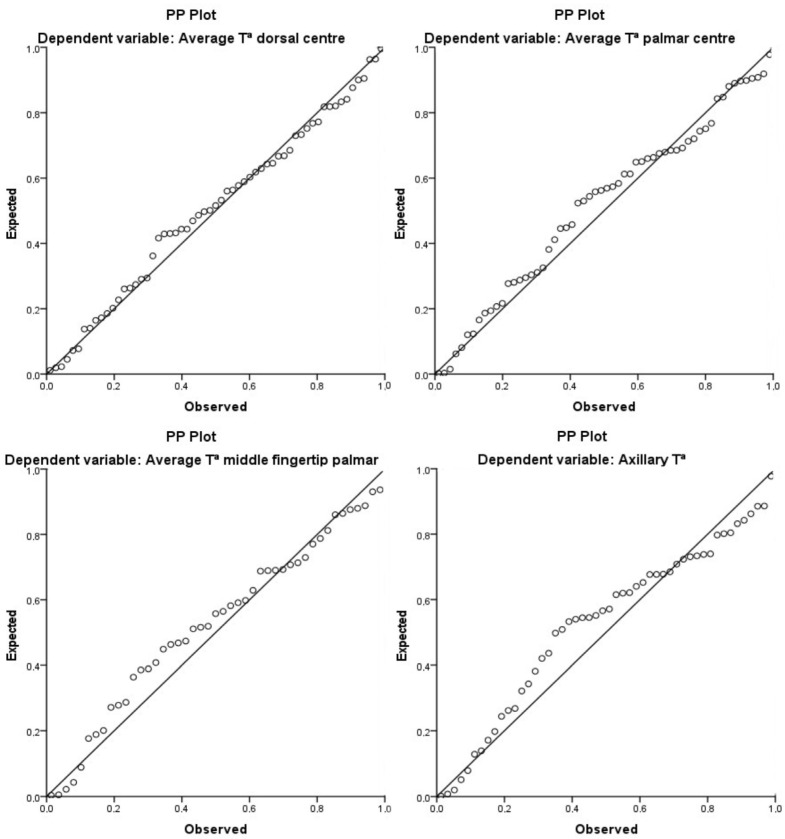
Linear regression standardized P-P plot of predictive factors associated with peripheral and central temperature in both populations.

The results of the multivariate regression analysis indicated that lysine was significantly associated with the dependent variable, the dorsal center average, and could predict approximately 21.5% of the total variance observed in healthy women (Table 2). Furthermore, in healthy women, when the palmar middle fingertip average and palmar center average were considered as dependent variables, the multivariate model demonstrated that serine and lysine were significantly associated with these dependent variables, predicting approximately 12.4% and 19% of the total variance, respectively (Table 2). Finally, when axillary temperature was employed as the dependent variable, citrulline was found to be significantly associated with the dependent variable, explaining approximately 20.2% of the total variance in healthy controls (Table 2). Figure 2 illustrates the principal correlations between amino acids and temperature variables.

## 3. Discussion

The prevalence of fibromyalgia syndrome (FMS) has increased markedly in recent years, reaching the status of a major public health concern [17]. The severity of FMS symptoms appears to be associated with a deficiency in FMS patients’ thermal regulation in response to ambient temperature fluctuations [4]. Thermal regulation in humans is a complex process in which blood-circulating macromolecules, such as amino acids, play an important role in peripheral vascular tone [12]. The present study aimed to examine the relationships between amino acid concentrations in the blood and peripheral and body core temperatures in individuals with and without FMS.

The first remarkable result is the different correlation patterns obtained in FMS patients and healthy controls between amino acid concentrations and thermographic variables. It is noteworthy that only one essential amino acid demonstrated a correlation with core or peripheral temperatures in the control group, whereas four essential amino acids exhibited a correlation with different body temperatures in FMS. This result may indicate that the thermal regulation process in FMS differs from that observed in healthy controls. Lys, Ser, and aminoadipic acid correlate with thermographic variables exclusively in healthy controls, but not FMS patients. Serine (Ser) is one of the most-studied amino acids related to body temperature regulation in animals. It is well established that Ser administration causes hypothermia [18]. A negative correlation between Ser blood concentration and thermographic variables of the hands was observed in the control group, but not in the FMS group, as evidenced by the results of our study. This finding suggests that the Ser function and metabolism may be altered in FMS patients. In this line, impairments in Ser metabolism have been associated with a number of peripheral and central nervous system syndromes [19]. Similarly, the concentration of lysine (Lys) and its metabolic precursor, aminoadipic acid, exhibit some correlations with several thermographic data in the hands of healthy controls. By contrast, only one variable in the FMS group showed a correlation. Furthermore, a negative correlation was observed between aminoadipic acid and axillary temperature exclusively in the control group. It has been demonstrated that deprivation of lysine in animals produces hyperthermia [20]. The results of our study corroborated this inverse correlation only in the healthy control group, whereas the correlation within the FMS group was positive. This suggests an alteration in the role of lysine in the thermal regulation of patients with FMS. Previous findings from our research group indicated a notable elevation in lysine levels in individuals with FMS compared to healthy controls [13]. It is noteworthy that alterations in lysine metabolism and function have been postulated in the context of hypothyroidism [21], a condition that presents symptoms analogous to those observed in FMS, including muscle pain, fatigue, and depression. Moreover, a recent meta-analysis indicated a correlation between thyroid disease and the onset of FMS [22].

On the other hand, the FMS group exhibited several positive correlations between the thermographic variables of the hands and glutamic acid, tryptophan, methionine, 3-methylhistidine, and tyrosine. Glutamic acid (Glu), or glutamate, is an excitatory amino acid that plays a pivotal role in thermal sensation and thermoregulation across a wide range of animals [23]. In animal models, a high intake of Glu results in disruptions to adaptive thermal regulation, leading to hypothermia [24]. In addition, Glu is associated with thermal hyperalgesia [25] and lower pain pressure thresholds in patients with FMS [26,27]. For this reason, our results suggest that Glu may play a key role in the thermoregulation process, which is closely associated with pain severity in FMS [28]. In our analysis, Glu and tryptophan (Trp) displayed the highest number of positive correlations with thermographic variables in individuals diagnosed with FMS. The effect of dietary and blood-circulating Trp on body temperature regulation has also been extensively demonstrated in a number of animal models [29,30]. An augmentation in Trp concentration has been demonstrated to result in an increase in serotonin production, which in turn triggers a hypothermic response [31]. However, our results show that higher blood Trp concentrations correlate positively with higher thermographic variables such as temperature in FMS patients and axillary mean temperature in healthy controls. In other words, elevated Trp concentrations are associated with higher peripheral temperature in FMS and body core temperature in healthy subjects. It is noteworthy that a disruption in tryptophan metabolism [32], elevated tryptophan degradation [33], and diminished serotonin levels have been observed in a cohort of FMS patients [34].

Additionally, methionine (Met) is also an essential amino acid that presents several positive correlations with thermographic variables exclusively within the FMS cohort. The administration of Met supplementation has demonstrated a protective role in animals exposed to heat stress [35,36] due to a hypothermic response [37]. In addition to Glu and Trp, our findings for Met suggest that these amino acids may not be associated with hypothermia in FMS patients. Moreover, if these amino acids have any function in FMS patients, it is to produce a hyperthermic response.

A positive correlation has been identified between the concentration of tyrosine (Tyr) and thermographic variables. Tyrosine (Tyr) is associated with the regulation of human body temperature. It has been demonstrated that supplementation with Tyr results in peripheral vasoconstriction, thereby maintaining core body temperature in older adults [38]. In contrast with this work, the positive correlations observed in our study indicated that elevated Tyr concentrations may provoke an increase in thermographic variables in FMS patients through the mechanism of peripheral vasodilation. This suggests an alteration in Tyr metabolism and/or function. A recent study has identified the blood Tyr concentration as a key biomarker for FMS diagnosis through machine learning [39]. It is noteworthy that Tyr is the metabolic precursor to dopamine and norepinephrine. Several studies have indicated that these two neuropeptides may play an important role in human thermoregulation [40,41] and in the development of the symptoms associated with FMS [42,43].

A derivative of histidine, 3-methylhistidine (3-MH), is mainly produced in skeletal muscle. In the present study, positive correlations were also identified between 3-MH and thermographic variables in the hands of FMS patients. The blood concentration of 3-MH has been employed as a biomarker of skeletal muscle protein turnover [44] as well as striated muscle toxicity and degradation [45]. An experimental model of FMS demonstrated increased protein turnover and skeletal muscle degradation [46], which is consistent with the elevated 3-MH levels observed in FMS blood compared to healthy controls [13]. It is crucial to emphasize the relationship between skeletal muscle function and both shivering and non-shivering thermoregulation [47].

Finally, the asparagine amino acid (Asn) exhibited positive correlations in the control and FMS groups. In the FMS group, significant correlations were observed between thermographic data from the dorsum of the hand and the Asn concentration in blood samples. By contrast, some weak correlations were found between Asn concentrations and thermographic variables in the palm of the hand in healthy subjects. It appears that Asn plays a role in the thermoregulatory processes observed in animals. Administering Asn to cell cultures has been demonstrated to enhance the thermogenic process in brown adipose cells, resulting in an elevated body temperature [48]. In the thermoregulatory process, the non-hairy skin (also referred to as glabrous skin), including the palms of the hands, functions as a radiator, emitting heat from the body to reduce the body’s core temperature (hyperthermia) in a warm environment. Nevertheless, the precise extent to which peripheral vasoconstriction of the skin affects body core temperature, and vice versa, remains unclear [49]. It is noteworthy that Asn levels are exclusively correlated with thermographic variables in the palm of the hands in the control group, whereas in FMS patients, they correlate with the dorsum of the hands. This result may indicate that Asn levels produce an effective response in the peripheral vasculature in the glabrous skin, thereby regulating body core temperature exclusively in the control group.

Considering all of the above, the cluster of circulating amino acids associated with thermoregulation in FMS differs from that observed in healthy controls, indicating a distinct pathway for body core temperature control in FMS. Given the established relationship between pain and thermoregulation in some conditions [50], including FMS, the present study provides preliminary support for the hypothesis that a nutritional intervention to control amino acid homeostasis may be a potential method for managing symptoms associated with FMS. In this regard, a recent review suggested that nutritional interventions should be considered in treating patients with FMS [51].

The results of this work should be interpreted in the context of its own limitations. The sample in our study was very small and comprised a specific population of FMS women. Therefore, our data might not be generalizable to other populations. It is possible that intra-subject variability exists in the thermographic record [52]. In order to reduce this potential bias, we followed the recommendations set out by the European Association of Thermography (EAT). It is important to clarify that our study was conducted within the human thermoneutral zone. Furthermore, our results are difficult to discuss due to limited available knowledge regarding the specific role of amino acids in the human thermoregulatory process. Moreover, the physiological mechanisms underlying thermal regulation remain poorly understood. In response to exposure to cool/warm conditions, a peripheral vasoconstriction/vasodilation response is initiated to reduce or increase heat loss from the extremities, regulating the body’s core temperature. Nevertheless, the scientific literature has not yet identified the magnitude of this relationship between peripheral and central thermoregulation responses. To elucidate the role of amino acids in human thermoregulation, future studies with a larger sample size should corroborate our results by studying the association between amino acids and body temperatures in subjects exposed to warm or cool environments. Finally, no inferences about causal relationships can be made due to the cross-sectional design of the study.

## 4. Materials and Methods

### 4.1. Study Design and Participants

We conducted this observational case–control study between September 2019 and March 2020. The study was carried out in accordance with the Declaration of Helsinki (modified in 2013) of the World Medical Association (WMA) and received approval from the Ethics Committee on Human Research (CEIH) of the University of Granada (Spain) (approval number: 1718-N-18). A total of forty-seven patients with FMS were contacted both via the Association of Fibromyalgia of Granada (AGRAFIM, Spain) and the Association of Fibromyalgia of Jaén (AFIXA, Spain). In total, fifty-nine healthy subjects were enrolled in this study. The healthy subjects were recruited from friends, relatives, and colleagues, as well as from the Faculty of Health Sciences staff (University of Granada, Spain).

The patients with FMS were aged between 30 and 70 years old and previously diagnosed with FMS by a professional rheumatologist of the Public Health System of Andalucía (Spain) in accordance with the 2016 ACR criteria for FMS [6]. The exclusion criteria for both FMS patients and healthy controls were as follows: (1) male sex; (2) grade II obesity (body mass index (BMI) ≥ 35 kg/m^2^); (3) presence of rheumatic diseases, diabetes mellitus, or cancer; (4) presence of cardiac, renal, or hepatic insufficiency; (5) severe physical disability or psychiatric illness; (6) skin disorders; (7) previous history of surgery; (8) pregnancy or lactation; (9) treatment with vasoactive drugs, anticoagulants, corticosteroids, estrogens, or agonist/antagonist of opioid receptors (morphine, tramadol, oxycodone, naltrexone, etc.). Given the observed differences in the concentration of circulating amino acids between men and women [53] and the higher prevalence of FMS in women compared to men, male patients were excluded from our study to avoid a possible confounder. No financial incentive was provided to the participants, and all provided written informed consent.

### 4.2. Measures

The demographic data of the participants were obtained via interviews and questionnaires, which included information regarding age, height, weight, BMI, duration of FMS, menopause status, dominant hand, and drug history. Participants provided these data during their first visit to our laboratory at the Faculty of Health Sciences at the University of Granada (Spain). Subsequently, the participants were scheduled for a second visit to our laboratory, during which the measurements of central and peripheral temperature, as well as blood extractions, were carried out.

#### 4.2.1. Peripheral Skin Temperature and Central Temperature Assessments

Prior to assessing both peripheral skin and central temperatures, women with FMS were instructed to wear comfortable clothing and refrain from wearing accessories such as watches, bracelets, or rings on their hands to ensure the accuracy of the readings. Furthermore, participants were asked to refrain from engaging in physical exertion and abstain from consuming food or vasoactive substances (alcohol, caffeine, tea, or nicotine) for 2 h prior to the evaluation. Before the acquisition of thermographic variables, patients remained seated in a comfortable position for 20 min in a climate-controlled room, without direct ventilation, at 24 °C in 50–60% humidity. These conditions were in compliance with the recommendations of the EAT [54].

**Figure 3:**
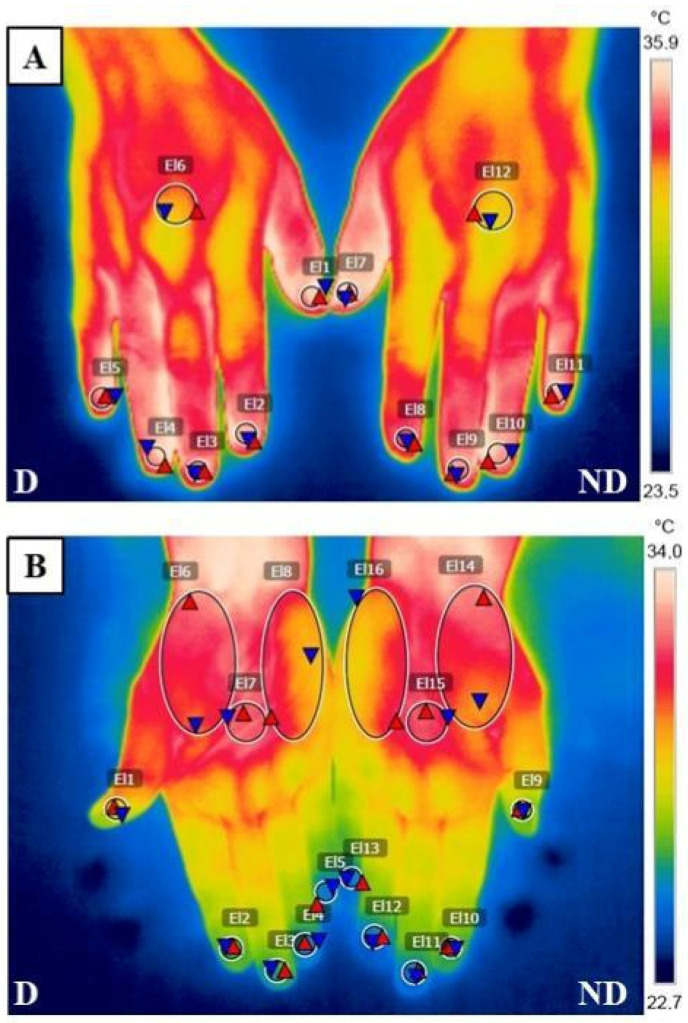
Thermograms of the hands in a patient with fibromyalgia and in a healthy woman. (**A**) Thermographic image of the dorsal area of both hands of a woman diagnosed with fibromyalgia (El1 and El7 = thumb finger; El2 and El8 = index finger; El3 and El9 = middle finger; El4 and El10 = ring finger; El5 and El11 = pinkie finger; El6 and El12 = dorsal centre). (**B**) Thermographic image of the palmar area of both hands of a healthy woman (El1 and El9 = thumb finger; El2 and El10 = index finger; El3 and El11 = middle finger; El4 and El12 = ring finger; El5 and El13 = pinkie finger; El6 and El14 = thenar eminence; El7 and El15 = palmar centre; El8 and El16 = hipothenar eminence). Red triangles = the point of maximum temperature within each area. Blue triangles = the point of minimum temperature within each area.

The peripheral skin temperature of the hands was recorded using infrared thermography (IRT), a non-invasive technique that provides information on local changes in microvascularization and blood flow within the skin tissue [55]. A calibrated FLIR B335 infrared thermographic camera (FLIR Systems, Inc., Täby, Sweden) was employed for the acquisition of thermographic images of the dorsal and palmar skin areas of the hands, characterized by a resolution of 320 × 240 pixels, a frequency of 30 Hz, an object temperature range from −20 to 120 °C, an accuracy of ±2 °C, and a thermal sensitivity of 0.05 °C. All thermograms were obtained by the same clinician. This methodology was previously described in the scientific literature [56,57]. Figure 3 illustrates the temperature evaluations of the hands using FLIR Tools software (FLIR B335, FLIR Systems AB, Täby, Sweden).

A handheld infrared thermometer (Infrared Dermal Thermometers DT-1001-LN, Exergen Corporation, Watertown, MA, USA) was used to determine the temperature of the core body in both the external auditory canal and the armpit. The tympanic temperature accurately reflects the core temperature because of the relationship between the tympanic artery and the hypothalamus [58]. Additionally, the axillary temperature was also recorded as a reliable indicator of body temperature despite its lower sensitivity and specificity compared to the tympanic temperature measurement [59,60].

#### 4.2.2. Blood Samples Collection

Participants were instructed not to exert themselves physically on the day of blood extraction. The blood samples were collected by the same expert practitioner from the antecubital vein into an anticoagulant-free tube (BD Vacutainer™ Venous Blood Collection Tubes: SST™ Serum Separation Tubes, Thermo Fisher, Waltham, MA, USA) at the same time of day (between 8 and 9 am) and after an overnight fast. Blood was allowed to clot for 30 min at room temperature. Each tube was then centrifuged at 3500 rpm for 5 min at 4 °C to obtain serum samples (Allegra Centrifuge, Beckman Coulter, Indianapolis, IN, USA). The serum samples were then divided into aliquots and stored at −80 °C until required for analysis.

### 4.3. Circulating Amino Acids Determination and Calculations

The concentration of circulating amino acids in the serum samples was determined with a high-performance liquid chromatography system (HPLC) coupled to a fluorescence detector following the protocol described by Ramírez-Expósito et al. [61]. In this protocol, the serum samples were subjected to deproteination through ultrafiltration, employing a 10,000 molecular weight cutoff filter. Afterward, the deproteinated serum was precolumn derivatized with OPA reagent (o-phthaldialdehyde in borate buffer pH 9.5 containing 3-mercaptopropionic acid) and introduced through a refrigerated Perkin–Elmer Series 200 automatic sample injector into a 150 × 3.9 mm Waters Resolve 5 μ C-18 column. After separating the samples into several phases inside the HPLC system, these phases were automatically introduced to the fluorescence detector (Perkin–Elmer Series 200a, PerkinElmer, Waltham, MA, USA), which was set at an excitation wavelength of 340 nm and an emission wavelength of 420 nm. Data were processed with TotalChrom WorkStation version 6.3.1 software from Perkin–Elmer. Concentrations were expressed in picomoles of amino acids (AAs) per microliter (pmoles/μL).

Furthermore, the amino acids were classified into groups based on their chemical characteristics. The concentrations of Val, Leu, and Ile were combined to form a single variable representing branched-chain amino acids (BCAAs). The BCAAs, in conjunction with the aromatic AAs Tyr and Phe, were aggregated as large neutral AAs (LNAAs). The BCAAs, in conjunction with Phe, Met, Trp, Thr, Lys, and His, were summed up as essential AAs (EAAs). Ala, Gly, Ser, Gln, Arg, Tau, Glu, Asn, Asp, Orn, and Cit were aggregated as non-essential AAs (NEAAs). Basic AAs (BAAs) were calculated as the sum of Arg, Orn, Lys, and His.

### 4.4. Statistical Analysis

The required sample size was calculated using G*Power Version 3.1.9.7 software (Heinrich Heine University Düsseldorf, Germany). Given the results of previous studies on the relationship between serum glutamate concentrations and the clinical picture in patients with FMS [62], it was determined that a minimum of 12 subjects per group would be required to achieve a power of 80% and a significance level (α error) of 0.05.

The statistical analysis of the data was conducted using SPSS Statistics Version 24 © software for Windows (IBM Corporation, Armonk, NY, USA). The normality of the data distribution and the homogeneity of variance were assessed using the Kolmogorov–Smirnov and the Levene tests, respectively. To compare differences between FMS and healthy women, an unpaired Student’s *t*-test with a 95% confidence interval (95% CI; α = 0.05) was applied for continuous variables, while the chi-square test (χ^2^) was used for categorical variables. The associations between amino acid concentrations and the peripheral skin temperature of the hands, as well as tympanic and axillary temperature, were examined using a linear regression analysis test with adjustments made for age, menopause status, and body mass index (BMI) [63,64,65]. Since there were no differences between dominant and non-dominant sides, the unified average value of both hands was calculated for key variables. Subsequently, Pearson’s bivariate correlation analysis was performed to evaluate the relationship between circulating amino acids and peripheral and central temperature in FMS and healthy women groups. Finally, a multivariate regression analysis was carried out. After the collinearity analysis, tryptophan, 3-methylhistidine, lysine, serine, aminoadipic acid, asparagine, and citrulline were included as independent variables, and the middle fingertip at the dorsal site, dorsal center, middle fingertip at the palmar site, palmar center, and axillary temperature as dependent variables. The data were reported as beta estimates (β) with 95% CIs and *p*-values. The level of statistical significance was set at *p* < 0.05.

## 5. Conclusions

In consideration of the findings, several correlations were identified between circulating amino acids and the peripheral temperature of the skin in the hand areas of patients with FMS and in healthy controls. Nevertheless, these observed correlation patterns diverge markedly between patients with FMS and healthy controls. This finding suggests a potential alteration in the role of circulating amino acids in the thermoregulatory process in patients with FMS.

Further studies with a larger sample size are required to clarify the role of circulating amino acids in thermoregulation in patients with FMS.

## Figures and Tables

**Table 1:** Demographic data, central temperature, and serum-free amino acid concentrations in women with fibromyalgia and healthy women.

	Women with FMS (*n* = 47)	Healthy Women (*n* = 59)	
Variable	Mean ± SD/Frequency (%)	Mean ± SD/Frequency (%)	*p*-Value
Age (years)	54.94 ± 6.59	53.47 ± 8.50	0.335
Height (cm)	159.26 ± 5.61	159.24 ± 5.77	0.987
Weight (kg)	66.28 ± 7.26	64.10 ± 7.86	0.146
BMI (kg/cm^2^)	26.19 ± 3.18	25.36 ± 3.55	0.210
Age of menarche (years)	12.42 ± 1.54	13.35 ± 4.22	0.202
Age of onset of menopause (years)	48.50 ± 5.14	49.61 ± 3.73	0.323
Duration of FMS (years)	11.29 ± 7.82	-	-
Menopause status			
Pre-menopausal	34 (72.34)	34 (57.63)	0.117
Post-menopausal	13 (27.66)	25 (42.37)	
FIQ-R			
FIQ-R.1	20.01 ± 6.11	-	-
FIQ-R.2	13.70 ± 5.22	-	-
FIQ-R.3	38.34 ± 7.56	-	-
Total score	72.02 ± 16.93	-	-
VAS (mm)	73.62 ± 18.58	19.66 ± 22.01	<0.001 *
Tympanic temperature °C	36.11 ± 0.59	35.80 ± 0.72	0.023 *
Axillary temperature °C	35.23 ± 0.89	35.57 ± 0.83	0.074
Serum-free amino acids (pmoles/µL)			
Phenylalanine	73.41 ± 28.35	54.59 ± 7.32	<0.001 *
Methionine	27.84 ± 16.30	20.68 ± 2.09	0.001 *
Tryptophan	97.00 ± 69.33	71.24 ± 13.14	0.006 *
Threonine	139.79 ± 89.39	92.51 ± 4.75	<0.001 *
Lysine	129.54 ± 75.54	113.60 ± 6.35	0.109
Histidine	43.48 ± 23.07	28.80 ± 0.98	<0.001 *
Alanine	232.85 ± 88.15	320.75 ± 18.52	<0.001 *
Glycine	76.18 ± 34.17	52.92 ± 12.44	<0.001 *
Serine	134.39 ± 65.54	118.34 ± 8.44	0.065
Glutamine	151.08 ± 47.40	133.26 ± 5.58	0.005 *
Arginine	69.62 ± 24.92	61.33 ± 5.10	0.014 *
Taurine	84.88 ± 33.51	61.43 ± 12.99	<0.001 *
Tyrosine	67.26 ± 31.54	55.88 ± 8.27	0.009 *
Glutamic acid	70.07 ± 31.59	37.51 ± 3.29	<0.001 *
Asparagine	48.33 ± 22.76	39.30 ± 2.49	0.003 *
Aspartic acid	30.91 ± 14.23	21.72 ± 3.15	<0.001 *
Ornithine	469.92 ± 245.55	361.00 ± 68.76	0.002 *
Citrulline	25.86 ± 12.57	24.47 ± 5.05	0.441
Aminoadipic acid	1.14 ± 1.03	0.64 ± 0.35	0.001 *
Carnosine	165.26 ± 107.35	168.54 ± 31.53	0.824
γ-aminobutyric acid (GABA)	205.12 ± 66.28	215.57 ± 40.51	0.320
Isoleucine	67.94 ± 36.49	47.25 ± 8.65	<0.001 *
Leucine	170.62 ± 91.00	115.24 ± 24.53	<0.001 *
Valine	249.91 ± 142.03	188.52 ± 5.69	0.001 *
3-Methylhistidine	148.42 ± 101.49	82.58 ± 12.92	<0.001 *
5-Methylhistidine	3.31 ± 2.59	2.41 ± 0.95	0.015 *

Data are expressed as mean ± standard deviation (SD) for quantitative variables and as frequency (%) for qualitative variables. Abbreviations: FMS—fibromyalgia syndrome; BMI—body mass index; FIQ-R—revised Fibromyalgia Impact Questionnaire; FIQ-R.1—activity level of FIQ-R; FIQ-R.2—overall impact of the FIQ-R; FIQ-R.3—intensity of symptoms of FIQ-R; VAS—visual analog scale; °C—degrees Celsius; pmoles/µL—picomoles of amino acid per microliter. * Significance level *p* < 0.05.

**Table 2:** Final multiple regression model of predictive associated factors to peripheral temperature of the hands and core body temperature in women with fibromyalgia and healthy women.

**Women with FMS (*n* = 47)**						
**Middle Fingertip Average at the Dorsal Site of the Hands (°C) (r^2^ = 0.140)**						
		**95% CI**				
**Independent Variables**	**B**	**Lower Limit**	**Upper Limit**	**β**	**SE**	***p*** **-Value**
Tryptophan (pmoles/µL)	0.010	−0.002	0.022	0.242	0.006	0.104
3-Methylhistidine (pmoles/µL)	0.007	−0.001	0.015	0.244	0.004	0.101
**Healthy Women (*n* = 59)**						
**Dorsal Center Average (°C) (r^2^ = 0.215)**						
		**95% CI**				
**Independent Variables**	**B**	**Lower Limit**	**Upper Limit**	**β**	**SE**	***p*** **-Value**
Lysine (pmoles/µL)	−0.077	−0.135	−0.019	−0.321	0.029	0.010 *
Serine (pmoles/µL)	−0.036	−0.080	0.007	−0.202	0.022	0.097
Aminoadipic acid (pmoles/µL)	−0.963	−2.000	0.075	−0.224	0.518	0.068
**Women with FMS (*n* = 47)**						
**Middle Fingertip Average at the Palmar Site of the Hands (°C) (r^2^ = 0.128)**						
		**95% CI**				
**Independent Variables**	**B**	**Lower Limit**	**Upper Limit**	**β**	**SE**	***p*** **-Value**
Tryptophan (pmoles/µL)	0.011	−0.001	0.023	0.266	0.006	0.077
3-Methylhistidine (pmoles/µL)	0.005	−0.003	0.014	0.194	0.004	0.194
**Healthy Women (*n* = 59**)						
**Middle Fingertip Average at the Palmar Site of the Hands (°C) (r^2^ = 0.124)**						
		**95% CI**				
**Independent Variables**	**B**	**Lower Limit**	**Upper Limit**	**β**	**SE**	***p*** **-Value**
Serine (pmoles/µL)	−1.113	−0.222	−0.004	−0.261	0.054	0.043 *
Asparagine (pmoles/µL)	0.337	−0.038	0.713	−0.227	0.187	0.077
**Healthy Women (*n* = 59)**						
**Palmar Center Average (°C) (r^2^ = 0.190)**						
		**95% CI**				
**Independent Variables**	**B**	**Lower Limit**	**Upper Limit**	**β**	**SE**	***p*** **-Value**
Lysine (pmoles/µL)	−0.077	−0.128	−0.025	−0.361	0.026	0.005 *
Serine (pmoles/µL)	−0.034	−0.072	0.004	−0.216	0.019	0.081
**Healthy Women (*n* = 59)**						
**Axillary Temperature (°C) (r^2^ = 0.202)**						
		**95% CI**				
**Independent Variables**	**B**	**Lower Limit**	**Upper Limit**	**β**	**SE**	***p*** **-Value**
Tryptophan (pmoles/µL)	0.012	−0.004	0.028	0.196	0.008	0.149
Citrulline (pmoles/µL)	0.052	0.008	0.095	0.315	0.022	0.021 *
Aminoadipic acid (pmoles/µL)	−0.547	−1.161	0.068	−0.239	0.305	0.080

* Significance level *p* < 0.05. Abbreviations: FMS—fibromyalgia syndrome; °C—celsius degree; r^2^—regression coefficient of determination; 95% CI—95% confidence interval; pmoles/µL—picomoles of amino acid per microliter; B—regression coefficient; β—adjusted coefficient from multiple linear regression analysis; SE—coefficient standard error.

## Data Availability

The datasets generated during the current study are available from the corresponding author on reasonable request.

## Supplementary Materials

The following supporting information can be downloaded at: https://www.mdpi.com/article/10.3390/ijms252413517/s1.
