# Supplementary material for: Different Correlation Patterns Between Circulating Amino Acids and Body Temperature in Fibromyalgia Syndrome: A Cross-Sectional Study
Source: Int J Mol Sci. 2024 Dec 17;25(24):13517. doi: 10.3390/ijms252413517 (PMC11676748; doi:10.3390/ijms252413517)
Supplement: Supplementary file 1 [file ijms-25-13517-s001.zip › ijms-3337485-supplementary.pdf]

Table S1. Correlations between serum free amino acids and temperature of the dorsal site of both hands in women with Fibromyalgia and controls.

	Serum free amino acids (pmoles/µL)											
	Phenylalanine			Methionine			Tryptophan			Threonine		
**Variable**	**β**	**95 % CI**	***p*-value**	**β**	**95 % CI**	***p*-value**	**β**	**95 % CI**	***p*-value**	**β**	**95 % CI**	***p*-value**
**Dorsal sites of both hands**												
**Thumb fingertip average (ºC)**												
Women with FMS												
D	1.600	(-1.711,4.912)	0.334	**2.222**	**(0.401,4.042)**	**0.018***	**8.906**	**(1.207,16.604)**	**0.024***	3.188	(-7.332,13.708)	0.544
ND	1.399	(-1.964,4.762)	0.405	**2.101**	**(0.239,3.962)**	**0.028***	**8.586**	**(0.741,16.431)**	**0.033***	2.912	(-7.750,13.574)	0.584
Healthy women												
D	-0.025	(-0.596,0.547)	0.931	-0.002	(-0.167,0.162)	0.977	-0.166	(-1.145,0.813)	0.736	0.115	(-0.254,0.485)	0.534
ND	0.106	(-0.508,0.720)	0.730	0.013	(-0.163,0.190)	0.880	-0.165	(-1.218,0.888)	0.755	0.049	(-0.349,0.448)	8.06
**Index fingertip average (ºC)**												
Women with FMS												
D	1.522	(-1.532,4.576)	0.320	**2.153**	**(0.486,3.821)**	**0.013***	**8.450**	**(1.371,15.529)**	**0.021***	4.970	(-4.656,14.597)	0.303
ND	1.193	(-1.930,4.316)	0.445	**1.867**	**(0.132,3.603)**	**0.036***	**7.955**	**(0.678,15.231)**	**0.033***	4.781	(-5.026,14.588)	0.330
Healthy women												
D	-0.018	(-0.545,0.510)	0.947	-0.013	(-0.165,0.138)	0.861	-0.243	(-1.145,0.659)	0.591	0.112	(-0.229,0.453)	0.512
ND	0.175	(-0.399,0.749)	0.543	-0.006	(-0.172,0.159)	0.938	-0.103	(-1.090,0.885)	0.836	0.052	(-0.322,0.425)	0.783
**Middle fingertip average (ºC)**												
Women with FMS												
D	1.403	(-1.726,4.532)	0.370	**2.007**	**(0.278,3.735)**	**0.024***	**7.548**	**(0.190,14.905)**	**0.045***	4.044	(-5.842.13.931)	0.413
ND	0.897	(-2.305,4.099)	0.574	1.672	(-0.125,3.469)	0.067	7.220	(-0.310,14.749)	0.060	2.618	(-7.488,12.725)	0.603
Healthy women												
D	-0.037	(-0.553,0.480)	0.887	0.005	(-0.143,0.154)	0.944	-0.236	(-1.120,0.647)	0.594	0.117	(-0.217,0.450)	0.486
ND	0.171	(-0.408,0.750)	0.556	0.009	(-0.158,0.176)	0.914	-0.017	(-1.013,0.980)	0.973	0.103	(-0.272,0.479)	0.583
**Ring fingertip average (ºC)**												
Women with FMS												
D	1.280	(-1.862,4.422)	0.415	**1.869**	**(0.120,3.618)**	**0.037***	**7.541**	**(0.163,14.919)**	**0.045***	3.466	(-6.468,13.400)	0.485
ND	0.603	(-2.661,3.866)	0.711	1.511	(-0.334,3.355)	0.106	6.967	(-0.725,14.660)	0.075	1.965	(-8.329,12.258)	0.702
Healthy women												
D	-0.009	(-0.526,0.508)	0.973	-0.001	(-0.150,0.147)	0.985	-0.202	(-1.087,0.683)	0.649	0.130	(-0.203,0.464)	0.437
ND	0.184	(-0.381,0.750)	0.516	-0.004	(-0.167,0.160)	0.965	-0.063	(-1.036,0.911)	0.898	0.084	(-0.283,0.452)	0.647

Table S1. *Cont.*

Variable	Phenylalanine			Methionine			Tryptophan			Threonine		
	**Serum free amino acids (pmoles/µL)**											
	**β**	**95 % CI**	***p*-value**	**β**	**95 % CI**	***p*-value**	**β**	**95 % CI**	***p*-value**	**β**	**95 % CI**	***p*-value**
**Dorsal sites of both hands**												
**Pinkie fingertip average (ºC)**												
Women with FMS												
D	1.081	(-2.109,4.272)	0.497	1.777	(-0.007,3.560)	0.051	**7.745**	**(0.282,15.207)**	**0.042***	4.329	(-5.700,14.358)	0.388
ND	0.859	(-2.377,4.096)	0.595	1.608	(-0.216,3.431)	0.082	**7.916**	**(0.372,15.460)**	**0.040***	2.024	(-8.202,12.249)	0.691
Healthy women												
D	0.006	(-0.504,0.517)	0.980	-0.027	(-0.173,0.120)	0.716	-0.202	(-1.077,0.672)	0.644	0.180	(-0.148,0.508)	0.276
ND	0.200	(-0.354,0.753)	0.473	-0.008	(-0.168,0.152)	0.921	-0.010	(-0.963,0.944)	0.984	0.095	(-0.265,0.454)	0.600
**Dorsal centre average (ºC)**												
Women with FMS												
D	2.023	(-4.575,8.621)	0.539	**4.809**	**(1.260,8.357)**	**0.009***	**20.388**	**(5.512,35.264)**	**0.008***	12.850	(-7.657,33.356)	0.213
ND	0.734	(-5.158,6.627)	0.802	**3.741**	**(0.516,6.966)**	**0.024***	**17.017**	**(3.634,30.401)**	**0.014***	6.709	(-11.770,25.188)	0.467
Healthy women												
D	-0.824	(-2.092,0.444)	0.198	0.017	(-0.354,0.387)	0.928	-0.238	(-2.446,1.970)	0.830	0.017	(-0.818,0.853)	0.968
ND	-0.674	(-1.997,0.650)	0.312	0.116	(-0.267,0.499)	0.547	0.469	(-1.819,2.758)	0.683	-0.002	(-0.869,0.865)	0.996

Table S1. *Cont.*

	Serum free amino acids (pmoles/µL)											
	Lysine			Histidine			Alanine			Glycine		
**Variable**	**β**	**95 % CI**	***p*-value**	**β**	**95 % CI**	***p*-value**	**β**	**95 % CI**	***p*-value**	**β**	**95 % CI**	***p*-value**
**Dorsal sites of both hands**												
**Thumb fingertip average (ºC)**												
Women with FMS												
D	6.914	(-1.712,15.541)	0.113	2.224	(-0.396,4.844)	0.094	-2.278	(-11.416,6.861)	0.617	1.504	(-2.415,5.422)	0.443
ND	5.372	(-3.480,14.225)	0.227	1.920	(-0.760,4.599)	0.155	-4.880	(-14.031,4.217)	0.288	1.708	(-2.252,5.669)	0.389
Healthy women												
D	-0.358	(-0.838,0.123)	0.141	0.018	(-0.056,0.092)	0.631	1.087	(-0.331,2.504)	0.130	0.490	(-0.475,1.455)	0.313
ND	-0.479	(-0.990,0.032)	0.066	0.022	(-0.058,0.101)	0.588	1.008	(-0.526,2.541)	0.193	0.605	(-0.430,1.641)	0.246
**Index fingertip average (ºC)**												
Women with FMS												
D	6.495	(-1.459,14.449)	0.107	2.232	(-0.170,4.634)	0.068	-1.241	(-9.695,7.212)	0.768	1.698	(-1.906,5.302)	0.347
ND	4.999	(-3.209,13.208)	0.226	2.133	(-0.324,4.589)	0.087	-2.774	(-11.337,5.789)	0.516	1.700	(-1.968,5.367)	0.354
Healthy women												
D	-0.334	(-0.786,0.099)	0.125	0.034	(-0.034,0.102)	0.320	0.834	(-0.482,2.151)	0.209	0.299	(-0.596,1.195)	0.505
ND	-0.434	(-0.913,0.046)	0.076	0.028	(-0.046,0.102)	0.452	1.106	(-0.323,2.534)	0.127	0.666	(-0.300,1.631)	0.173
**Middle fingertip average (ºC)**												
Women with FMS												
D	6.125	(-2.045,14.294)	0.138	1.992	(-0.488,4.473)	0.112	-2.640	(-11.247,5.967)	0.539	1.802	(-1.878,5.481)	0.328
ND	4.205	(-4.233,12.643)	0.320	1.741	(-0.803,4.286)	0.174	-4.029	(-12.730,4.672)	0.355	1.478	(-2.281,5.236)	0.431
Healthy women												
D	-0.369	(-0.801,0.062)	0.092	0.027	(-0.040,0.093)	0.429	1.053	(-0.224,2.330)	0.104	0.399	(-0.475,1.273)	0.364
ND	-0.441	(-0.925,0.043)	0.073	0.036	(-0.039,0.111)	0.343	1.152	(-0.286,2.590)	0.114	0.620	(-0.356,1.596)	0.209
**Ring fingertip average (ºC)**												
Women with FMS												
D	5.557	(-2.675,13.789)	0.180	1.932	(-0.559,4.424)	0.125	-3.058	(-11.672,5.557)	0.477	1.286	(-2.425,4.997)	0.488
ND	3.429	(-5.191,12.048)	0.426	1.478	(-1.128,4.084)	0.258	-5.018	(-13.818,3.781)	0.256	1.306	(-2.523,5.135)	0.495
Healthy women												
D	-0.344	(-0.777,0.089)	0.117	0.023	(-0.044,0.090)	0.490	0.941	(-0.344,2.225)	0.148	0.480	(-0.392,1.351)	0.275
ND	-0.437	(-0.909,0.036)	0.069	0.032	(-0.041,0.105)	0.386	1.101	(-0.307,2.508)	0.123	0.739	(-0.208,1.686)	0.124

Table S1. *Cont.*

Variable	Serum free amino acids (pmoles/µL)											
	Lysine			Histidine			Alanine			Glycine		
	**β**	**95 % CI**	***p*-value**	**β**	**95 % CI**	***p*-value**	**β**	**95 % CI**	***p*-value**	**β**	**95 % CI**	***p*-value**
**Dorsal sites of both hands**												
**Pinkie fingertip average (ºC)**												
Women with FMS												
D	5.079	(-3.295,13.453)	0.227	1.920	(-0.607,4.447)	0.132	-4.074	(-12.758,4.611)	0.349	1.510	(-2.241,5.261)	0.421
ND	3.837	(-4.708,12.382)	0.370	1.758	(-0.813,4.329)	0.175	-4.098	(-12.888,4.692)	0.352	1.408	(-2.393,5.209)	0.458
Healthy women												
D	-0.318	(-0.748,0.111)	0.143	0.025	(-0.041,0.091)	0.452	0.818	(-0.458,2.093)	0.204	0.546	(-0.312,1.404)	0.207
ND	-0.403	(-0.867,0.061)	0.088	0.033	(-0.039,0.104)	0.365	1.074	(-0.305,2.452)	0.124	0.734	(-0.193,1.661)	0.118
**Dorsal centre average (ºC)**												
Women with FMS												
D	**20.694**	**(4.362,37.027)**	**0.014***	**5.502**	**(0.427,10.577)**	**0.034***	-1.815	(-19.946,16.315)	0.841	5.051	(-2.592,12.695)	0.189
ND	13.966	(-1.059,28.991)	0.068	3.656	(-0.977,8.289)	0.119	-5.786	(-21.816,10.243)	0.470	4.667	(-2.120,11.454)	0.172
Healthy women												
D	**-1.309**	**(-2.355,-0.263)**	**0.015***	0.045	(-0.121,0.212)	0.588	-0.059	(-3.323,3.205)	0.971	0.278	(-1.918,2.473)	0.801
ND	**-1.327**	**(-2.415,-0.239)**	**0.018***	0.072	(-0.100,0.245)	0.405	1.477	(-1.886,4.839)	0.383	0.290	(-1.987,2.568)	0.799

Table S1. *Cont.*

Variable	Serum free amino acids (pmoles/µL)											
	**Serine**			**Glutamine**			**Arginine**			**Taurine**		
	**β**	**95 % CI**	***p*-value**	**β**	**95 % CI**	***p*-value**	**β**	**95 % CI**	***p*-value**	**β**	**95 % CI**	***p*-value**
**Dorsal sites of both hands**												
**Thumb fingertip average (ºC)**												
Women with FMS												
D	2.094	(-5.276,9.465)	0.569	1.939	(-3.569,7.448)	0.481	1.226	(-1.608,4.059)	0.387	-0.904	(-4.479,2.671)	0.612
ND	1.209	(-6.275,8.693)	0.746	2.098	(-3.475,7.670)	0.451	0.888	(-1.994,3.770)	0.537	-0.948	(-4.568,2.671)	0.599
Healthy women												
D	-0.512	(-1.163,0.138)	0.120	0.185	(-0.243,0.613)	0.389	-0.288	(-0.681,0.105)	0.148	-0.016	(-1.023,0.992)	0.975
ND	-0.647	(-1.341,0.046)	0.067	0.099	(-0.364,0.561)	0.671	-0.387	(-0.805,0.031)	0.069	-0.039	(-1.123,1.045)	0.942
**Index fingertip average (ºC)**												
Women with FMS												
D	2.012	(-4.789,8.814)	0.553	1.565	(-3.528,6.657)	0.538	1.522	(-1.073,4.117)	0.243	-0.661	(-3.966,2.643)	0.688
ND	1.504	(-5.429,8.437)	0.663	0.370	(-4.834,5.574)	0.886	0.843	(-1.830,3.515)	0.527	-0.949	(-4.303,2.406)	0.571
Healthy women												
D	**-0.630**	**(-1.219,-0.041)**	**0.037***	0.123	(-0.273,0.520)	0.535	-0.223	(-0.587,0.142)	0.227	0.238	(-0.690,1.165)	0.610
ND	**-0.762**	**(-1.399,-0.124)**	**0.020***	0.110	(-0.324,0.543)	0.614	-0.254	(-0.653,0.144)	0.206	0.001	(-1.016,1.015)	0.999
**Middle fingertip average (ºC)**												
Women with FMS												
D	1.486	(-5.479,8.452)	0.669	1.598	(-3.606,6.802)	0.538	1.352	(-1.312,4.015)	0.311	-0.950	(-4.320,2.420)	0.572
ND	0.966	(-6.128,8.061)	0.784	0.826	(-4.486,6.138)	0.755	0.496	(-2.244,3.235)	0.717	-1.032	(-4.458,2.393)	0.546
Healthy women												
D	**-0.611**	**(-1.188,-0.033)**	**0.039***	0.085	(-0.303,0.474)	0.662	-0.222	(-0.579,0.135)	0.219	0.129	(-0.781,1.039)	0.777
ND	**-0.756**	**(-1.400,-0.112)**	**0.022***	0.089	(-0.348,0.527)	0.684	-0.239	(-0.642,0.163)	0.239	0.051	(-0.973,1.076)	0.920
**Ring fingertip average (ºC)**												
Women with FMS												
D	1.276	(-5.712,8.263)	0.714	1.487	(-3.733,6.708)	0.568	1.072	(-1.611,3.755)	0.424	-0.952	(-4.331,2.426)	0.572
ND	0.088	(-7.133,7.309)	0.980	1.146	(-4.250,6.542)	0.670	0.334	(-2.455,3.123)	0.810	-1.167	(-4.647,2.313)	0.502
Healthy women												
D	-0.561	(-1.143,0.021)	0.059	0.053	(-0.337,0.442)	0.788	-0.258	(-0.614,0.098)	0.152	0.025	(-0.887,0.936)	0.957
ND	**-0.714**	**(-1.345,-0.082)**	**0.028***	0.086	(-0.341,0.514)	0.687	-0.266	(-0.658,0.125)	0.178	0.012	(-0.990,1.013)	0.982

Table S1. *Cont.*

	Serum free amino acids (pmoles/µL)											
	**Serine**			**Glutamine**			**Arginine**			**Taurine**		
**Variable**	**β**	**95 % CI**	***p*-value**	**β**	**95 % CI**	***p*-value**	**β**	**95 % CI**	***p*-value**	**β**	**95 % CI**	***p*-value**
**Dorsal sites of both hands**												
**Pinkie fingertip average (ºC)**												
Women with FMS												
D	0.299	(-6.789,7.387)	0.933	1.572	(-3.714,6.858)	0.551	1.012	(-1.709,3.732)	0.457	-1.611	(-5.008,1.786)	0.344
ND	0.434	(-6.739,7.607)	0.903	1.093	(-4.269,6.455)	0.682	0.298	(-2.473,3.069)	0.829	-1.175	(-4.631,2.282)	0.496
Healthy women												
D	-0.498	(-1.077,0.081)	0.090	0.056	(-0.329,0.441)	0.772	-0.238	(-0.590,0.114)	0.181	-0.076	(-0.976,0.825)	0.866
ND	**-0.660**	**(-1.282,-0.038)**	**0.038***	0.103	(-0.315,0.521)	0.624	-0.256	(-0.640,0.128)	0.187	-0.036	(-1.016,0.945)	0.942
**Dorsal centre average (ºC)**												
Women with FMS												
D	1.501	(-13.135,16.137)	0.837	2.072	(-8.876,13.020)	0.704	3.077	(-2.496,8.651)	0.271	-1.849	(-8.922,5.223)	0.600
ND	0.122	(-12.904,13.148)	0.985	-0.917	(-10.668,8.835)	0.850	2.306	(-2.674,7.286)	0.355	-2.494	(-8.757,3.769)	0.426
Healthy women												
D	-0.941	(-2.418,0.536)	0.207	0.460	(-0.503,1.423)	0.342	0.091	(-0.813,0.994)	0.841	-0.130	(-2.401,2.141)	0.909
ND	**-1.582**	**(-3.076,-0.087)**	**0.039***	0.600	(-0.394,1.594)	0.232	-0.001	(-0.938,0.937)	0.999	-0.412	(-2.766,1.942)	0.727

Table S1. *Cont.*

	Serum free amino acids (pmoles/µL)											
	**Glutamic acid**			**Asparagine**			**Aspartic acid**			**Ornithine**		
**Variable**	**β**	**95 % CI**	***p*-value**	**β**	**95 % CI**	***p*-value**	**β**	**95 % CI**	***p*-value**	**β**	**95 % CI**	***p*-value**
**Dorsal sites of both hands**												
**Thumb fingertip average (ºC)**												
Women with FMS												
D	**4.169**	**(0.736,7.602)**	**0.019***	2.181	(-0.346,4.707)	0.089	0.162	(-1.468,1.792)	0.842	8.761	(-19.542,37.065)	0.535
ND	**3.939**	**(0.429,7.450)**	**0.029***	1.916	(-0.666,4.498)	0.142	0.045	(-1.606,1.697)	0.956	11.174	(-17.404,39.751)	0.434
Healthy women												
D	0.158	(-0.092,0.408)	0.210	0.165	(-0.025,0.354)	0.087	0.048	(-0.199,0.95)	0.700	1.453	(-3.908,6.814)	0.589
ND	0.125	(-0.146,0.395)	0.360	0.173	(-0.031,0.376)	0.095	0.072	(-0.193,0.337)	0.589	2.038	(-3.717,7.793)	0.481
**Index fingertip average (ºC)**												
Women with FMS												
D	**4.278**	**(1.165,7.390)**	**0.008***	2.145	(-0.175,4.465)	0.069	0.185	(-1.319,1.690)	0.805	9.355	(-16.729,35.439)	0.473
ND	**3.899**	**(0.674,7.125)**	**0.019***	1.950	(-0.431,4.331)	0.106	0.172	(-1.359,1.703)	0.822	11.760	(-14.682,38.203)	0.374
Healthy women												
D	0.106	(-0.126,0.338)	0.365	0.138	(-0.037,0.313)	0.121	0.080	(-0.147,0.907)	0.482	1.759	(-3.177,6.696)	0.478
ND	0.131	(-0.122,0.385)	0.303	0.167	(-0.024,0.358)	0.085	0.062	(-0.186,0.311)	0.617	2.743	(-2.624,8.110)	0.310
**Middle fingertip average (ºC)**												
Women with FMS												
D	**3.985**	**(0.753,7.217)**	**0.017***	2.033	(-0.352,4.419)	0.093	0.022	(-1.517,1.560)	0.978	10.179	(-16.453,36.812)	0.444
ND	**3.452**	**(0.095,6.809)**	**0.044***	1.731	(-0.722,4.184)	0.162	0.069	(-1.496,1.634)	0.929	7.838	(-19.336,35.013)	0.563
Healthy women												
D	0.090	(-0.138,0.317)	0.434	0.152	(-0.019,0.322)	0.081	0.092	(-0.130,0.314)	0.412	1.477	(-3.364,6.318)	0.543
ND	0.073	(-0.184,0.330)	0.570	0.145	(-0.049,0.339)	0.139	0.073	(-0.117,0.324)	0.561	2.466	(-2.957,7.889)	0.366
**Ring fingertip average (ºC)**												
Women with FMS												
D	**3.766**	**(0.498,7.034)**	**0.025***	1.921	(-0.480,4.323)	0.114	0.063	(-1.480,1.605)	0.935	5.599	(-21.239,32.437)	0.676
ND	2.866	(-0.609,6.340)	0.103	1.349	(-1.171,3.870)	0.286	-0.006	(-1.598,1.585)	0.994	4.135	(-23.583,31.854)	0.765
Healthy women												
D	0.107	(-0.120,0.335)	0.350	0.142	(-0.029,0.314)	0.103	0.080	(-0.142,0.302)	0.474	2.167	(-2..658,6.993)	0.372
ND	0.109	(-0.141,0.360)	0.385	0.146	(-0.043,0.335)	0.127	0.076	(-0.168,0.321)	0.535	2.852	(-2.432,8.136)	0.284

Table S1. *Cont.*

	Serum free amino acids (pmoles/µL)											
	Glutamic acid			Asparagine			Aspartic acid			Ornithine		
**Variable**	**β**	**95 % CI**	***p*-value**	**β**	**95 % CI**	***p*-value**	**β**	**95 % CI**	***p*-value**	**β**	**95 % CI**	***p*-value**
**Dorsal sites of both hands**												
**Pinkie fingertip average (ºC)**												
Women with FMS												
D	**3.616**	**(0.283,6.949)**	**0.034***	1.510	(-0.953,3.974)	0.223	-0.028	(-1.590,1.534)	0.971	5.330	(-21.859,32.520)	0.694
ND	3.038	(-0.398,6.473)	0.081	1.514	(-0.980,4.008)	0.227	0.111	(-1.470,1.692)	0.888	4.343	(-23.194,31.879)	0.752
Healthy women												
D	0.104	(-0.121,0.329)	0.358	0.147	(-0.022,0.316)	0.086	0.054	(-0.166,0.275)	0.624	2.637	(-2.113,7.388)	0.271
ND	0.104	(-0.142,0.349)	0.401	0.143	(-0.042,0.328)	0.128	0.074	(-0.166,0.314)	0.538	2.279	(-2.915,7.473)	0.383
**Dorsal centre average (ºC)**												
Women with FMS												
D	**10.278**	**(3.773,16.782)**	**0.003***	**6.086**	**(1.279,10.893)**	**0.014***	-0.479	(-3.702,2.745)	0.766	21.828	(-34.014,77.671)	0.434
ND	**7.346**	**(1.305,13.388)**	**0.018***	**5.211**	**(0.910,9.513)**	**0.019***	-0.625	(-3.489,2.239)	0.661	15.815	(-33.988,65.619)	0.525
Healthy women												
D	0.117	(-0.453,0.688)	0.682	0.255	(-0.178,0.687)	0.243	0.207	(-0.348,0.761)	0.458	-1.840	(-13.944,10.264)	0.762
ND	0.071	(-0.522,0.664)	0.811	0.218	(-0.233,0.669)	0.337	0.136	(-0.440,0.713)	0.637	-3.264	(-15.803,9.275)	0.604

Table S1. *Cont.*

	Serum free amino acids (pmoles/µL)											
	Citrulline			Aminoadipic acid			Carnosine			γ-aminobutyric acid (GABA)		
**Variable**	**β**	**95 % CI**	***p*-value**	**β**	**95 % CI**	***p*-value**	**β**	**95 % CI**	***p*-value**	**β**	**95 % CI**	***p*-value**
**Dorsal sites of both hands**												
**Thumb fingertip average (ºC)**												
Women with FMS												
D	0.654	(-0.695,2.004)	0.333	0.040	(-0.067,0.147)	0.456	5.065	(-7.133,17.263)	0.406	3.261	(-4.373,10.895)	0.393
ND	0.657	(-0.710,2.024)	0.337	0.030	(-0.079,0.138)	0.583	2.979	(-9.444,15.403)	0.631	2.278	(-5.490,10.046)	0.557
Healthy women												
D	0.185	(-0.195,0.564)	0.333	-0.016	(-0.043,0.011)	0.250	1.409	(-1.009,3.828)	0.248	-0.517	(-3.711,2.676)	0.747
ND	0.179	(-0.230,0.588)	0.385	-0.013	(-0.043,0.016)	0.360	0.782	(-1.843,3.407)	0.553	-0.424	(-3.860,3.013)	0.806
**Index fingertip average (ºC)**												
Women with FMS												
D	0.807	(-0.427,2.041)	0.194	0.048	(-0.050,0.146)	0.330	6.736	(-4.417,17.888)	0.229	4.467	(-2.501,11.434)	0.203
ND	0.746	(-0.514,2.006)	0.238	0.043	(-0.057,0.143)	0.389	6.338	(-5.038,17.714)	0.267	3.426	(-3.726,10.578)	0.339
Healthy women												
D	0.216	(-0.132,0.565)	0.218	-0.017	(-0.042,0.008)	0.174	1.444	(-0.780,3.669)	0.199	-0.597	(-3.542,2.348)	0.686
ND	0.186	(-0.197,0.568)	0.335	-0.017	(-0.044,0.010)	0.221	0.859	(-1.598,3.316)	0.486	-0.728	(-3.944,2.489)	0.652
**Middle fingertip average (ºC)**												
Women with FMS												
D	0.783	(-0.481,2.047)	0.218	0.037	(-0.064,0.138)	0.463	6.512	(-4.908,17.932)	0.256	3.561	(-3.618,10.740)	0.322
ND	0.618	(-0.677,1.914)	0.340	0.021	(-0.082,0.124)	0.681	4.198	(-7.532,15.928)	0.474	2.495	(-4.854,9.845)	0.497
Healthy women												
D	0.175	(-0.168,0.518)	0.310	-0.017	(-0.041,0.008)	0.173	1.201	(-0.988,3.389)	0.276	-0.864	(-3.743,2.016)	0.550
ND	0.136	(-0.251,0.523)	0.484	-0.019	(-0.046,0.008)	0.167	0.722	(-1.760,3.203)	0.562	-1.146	(-4.381,2.089)	0.481
**Ring fingertip average (ºC)**												
Women with FMS												
D	0.735	(-0.535,2.005)	0.249	0.030	(-0.071,0.131)	0.551	5.512	(-5.991,17.014)	0.339	3.410	(-3.795,10.614)	0.345
ND	0.506	(-0.817,1.828)	0.444	0.012	(-0.093,0.117)	0.822	3.247	(-8.714,15.207)	0.586	2.725	(-4.742,10.192)	0.465
Healthy women												
D	0.184	(-0.158,0.527)	0.285	-0.016	(-0.040,0.009)	0.205	1.255	(-0.933,3.443)	0.255	-0.712	(-3.597,2.173)	0.623
ND	0.136	(-0.243,0.514)	0.475	-0.016	(-0.042,0.011)	0.250	0.567	(-1.861,2.994)	0.642	-0.729	(-3.899,2.441)	0.647

Table S1. *Cont.*

	Serum free amino acids (pmoles/µL)											
	**Citrulline**			**Aminoadipic acid**			**Carnosine**			**γ-aminobutyric acid (GABA)**		
**Variable**	**β**	**95 % CI**	***p*-value**	**β**	**95 % CI**	***p*-value**	**β**	**95 % CI**	***p*-value**	**β**	**95 % CI**	***p*-value**
**Dorsal sites of both hands**												
**Pinkie fingertip average (ºC)**												
Women with FMS												
D	0.725	(-0.563,2.013)	0.262	0.035	(-0.067,0.137)	0.492	5.782	(-5.859,17.422)	0.322	2.918	(-4.403,10.239)	0.425
ND	0.546	(-0.766,1.859)	0.405	0.012	(-0.092,0.116)	0.818	3.376	(-8.503,15.255)	0.569	2.712	(-4.707,10.130)	0.464
Healthy women												
D	0.200	(-0.138,0.537)	0.241	-0.014	(-0.039,0.010)	0.235	1.421	(-0.733,3.574)	0.192	-1.178	(-4.017,1.661)	0.409
ND	0.110	(-0.261,0.482)	0.554	-0.015	(-0.041,0.011)	0.263	0.475	(-1.904,2.854)	0.690	-0.750	(-3.854,2.354)	0.630
**Dorsal centre average (ºC)**												
Women with FMS												
D	1.736	(-0.909,4.381)	0.192	0.087	(-0.124,0.298)	0.409	20.318	(-3.418,43.783)	0.088	8.624	(-6.370,23.619)	0.252
ND	1.296	(-1.072,3.664)	0.275	0.023	(-0.166,0.212)	0.807	11.970	(-9.347,33.287)	0.263	6.193	(-7.223,19.609)	0.356
Healthy women												
D	0.392	(-0.464,1.248)	0.362	**-0.069**	**(-0.128,-0.010)**	**0.022***	4.909	(-0.444,10.262)	0.071	-0.714	(-7.916,6.488)	0.843
ND	0.329	(-0.562,1.219)	0.463	**-0.063**	**(-0.125,-0.001)**	**0.046***	2.835	(-2.839,8.508)	0.321	-1.109	(-8.579,6.361)	0.767

Table S1. *Cont.*

	Serum free amino acids (pmoles/µL)											
	**Isoleucine**			**Leucine**			**Valine**			**3-Methyl-histidine**		
**Variable**	**β**	**95 % CI**	***p*-value**	**β**	**95 % CI**	***p*-value**	**β**	**95 % CI**	***p*-value**	**β**	**95 % CI**	***p*-value**
**Dorsal sites of both hands**												
**Thumb fingertip average (ºC)**												
Women with FMS												
D	2.300	(-1.839,6.440)	0.268	7.892	(-2.438,18.223)	0.130	8.593	(-7.415,24.601)	0.284	**12.968**	**(1.892,24.044)**	**0.023***
ND	1.676	(-2.547,5.900)	0.427	7.138	(-3.445,15.643)	0.178	7.836	(-8.418,24.091)	0.336	**11.968**	**(0.620,23.316)**	**0.039***
Healthy women												
D	0.321	(-0.339,0.981)	0.333	0.521	(-1.237,2.279)	0.555	-0.037	(-0.483,0.408)	0.867	0.179	(-0.821,1.180)	0.721
ND	0.234	(-0.479,0.947)	0.513	0.462	(-1.431,2.354)	0.627	-0.029	(-0.508,0.451)	0.905	0.243	(-0.832,1.319)	0.652
**Index fingertip average (ºC)**												
Women with FMS												
D	2.208	(-1.608,6.024)	0.249	7.664	(-1.842,17.170)	0.111	7.701	(-7.089,22.490)	0.299	**11.759**	**(1.509,22.008)**	**0.026***
ND	1.660	(-2.252,5.573)	0.396	6.828	(-2.916,16.573)	0.164	6.568	(-8.539,21.675)	0.385	**10.058**	**(-0.573,20.688)**	**0.063***
Healthy women												
D	0.230	(-0.381,0.841)	0.453	-0.065	(-1.692,1.562)	0.937	-0.071	(-0.482,0.340)	0.732	-0.002	(-0.926,0.923)	0.997
ND	0.207	(-0.461,0.876)	0.536	0.197	(-1.580,1.974)	0.825	-0.042	(-0.491,0.407)	0.852	0.359	(-0.646,1.364)	0.477
**Middle fingertip average (ºC)**												
Women with FMS												
D	1.877	(-2.044,5.797)	0.339	7.185	(-2.580,16.951)	0.145	7.506	(-7.627,22.640)	0.322	**11.540**	**(1.011,22.069)**	**0.033***
ND	0.930	(-3.093,4.953)	0.643	5.382	(-4.676,15.439)	0.286	4.190	(-11.337,19.716)	0.589	10.408	(-0.441,21.256)	0.060
Healthy women												
D	0.268	(-0.329,0.865)	0.371	0.173	(-1.420,1.766)	0.828	-0.049	(-0.452,0.354)	0.807	-0.010	(-0.915,0.895)	0.982
ND	0.247	(-0.426,0.920)	0.465	-0.019	(-1.811,1.774)	0.983	-0.045	(-0.498,0.408)	0.844	0.245	(-0.771,1.261)	0.631
**Ring fingertip average (ºC)**												
Women with FMS												
D	1.553	(-2.392,5.498)	0.431	6.511	(-3.328,16.350)	0.189	5.426	(-9.836,20.688)	0.477	**11.263**	**(0.674,21.852)**	**0.038***
ND	0.184	(-3.917,4.286)	0.928	3.913	(-6.387,14.213)	0.447	1.159	(-14.684,17.002)	0.883	9.898	(-1.199,20.994)	0.079
Healthy women												
D	0.177	(-0.423,0.777)	0.556	0.098	(-1.497,1.693)	0.902	-0.060	(-0.463,0.343)	0.768	-0.010	(-0.916,0.896)	0.982
ND	0.199	(-0.460,0.859)	0.547	0.107	(-1.645,1.859)	0.903	-0.095	(-0.537,0.347)	0.668	0.344	(-0.647,1.335)	0.489

Table S1. *Cont.*

	Serum free amino acids (pmoles/μL)											
	**Isoleucine**			**Leucine**			**Valine**			**3-Methyl-histidine**		
**Variable**	**β**	**95 % CI**	***p*-value**	**β**	**95 % CI**	***p*-value**	**β**	**95 % CI**	***p*-value**	**β**	**95 % CI**	***p*-value**
**Dorsal sites of both hands**												
**Pinkie fingertip average (ºC)**												
Women with FMS												
D	1.360	(-2.644,5.364)	0.496	5.634	(-4.391,15.660)	0.263	4.594	(-10.895,20.082)	0.552	**11.252**	**(0.510,21.994)**	**0.041***
ND	0.712	(-3.357,4.781)	0.725	4.559	(-5.646,14.765)	0.372	2.010	(-13.722,17.742)	0.798	9.944	(-1.071,20.958)	0.076
Healthy women												
D	0.128	(-0.465,0.722)	0.666	-0.173	(-1.749,1.402)	0.826	-0.075	(-0.473,0.323)	0.708	0.027	(-0.868,0.922)	0.951
ND	0.192	(-0.453,0.838)	0.553	0.096	(-1.620,1.812)	0.911	-0.094	(-0.527,0.339)	0.665	0.439	(-0.529,1.406)	0.367
**Dorsal centre average (ºC)**												
Women with FMS												
D	6.657	(-1.386,14.699)	0.102	14.967	(-2.276,38.210)	0.080	20.235	(-11.245,51.716)	0.201	18.497	(-4.144,41.138)	0.107
ND	2.780	(-4.566,10.127)	0.449	10.369	(-8.053,28.790)	0.262	15.974	(-12.154,44.102)	0.258	17.901	(-2.113,37.914)	0.078
Healthy women												
D	0.476	(-1.019,1.970)	0.526	0.331	(-3.642,4.304)	0.868	-0.399	(-1.398,0.600)	0.426	-0.088	(-2.345,2.169)	0.938
ND	0.113	(-1.443,1.669)	0.885	0.020	(-4.104,4.144)	0.992	-0.709	(-1.734,0.316)	0.171	-0.353	(-2.693,1.987)	0.764

Table S1. *Cont.*

Variable	Serum free amino acids (pmoles/µL)					
	5-Methyl-histidine			Tyrosine		
	**β**	**95 % CI**	***p*-value**	**β**	**95 % CI**	***p*-value**
**Dorsal sites of both hands**						
**Thumb fingertip average (ºC)**						
Women with FMS						
D	0.063	(-0.246,0.372)	0.683	3.255	(-0.268,6.778)	0.069
ND	0.037	(-0.276,0.350)	0.811	3.141	(-0.440,6.723)	0.084
Healthy women						
D	0.034	(-0.037,0.104)	0.344	0.262	(-0.374,0.898)	0.413
ND	0.049	(-0.026,0.124)	0.199	0.206	(-0.480,0.892)	0.550
**Index fingertip average (ºC)**						
Women with FMS						
D	0.129	(-0.153,0.412)	0.361	**3.356**	**(0.139,6.573)**	**0.041***
ND	0.101	(-0.188,0.389)	0.485	2.919	(-0.402,6.240)	0.083
Healthy women						
D	0.030	(-0.035,0.095)	0.360	0.274	(-0.312,0.860)	0.352
ND	0.039	(-0.032,0.109)	0.280	0.154	(-0.490,0.798)	0.635
**Middle fingertip average (ºC)**						
Women with FMS						
D	0.111	(-0.178,0.401)	0.442	3.304	(0.004,6.605)	0.050
ND	0.033	(-0.264,0.329)	0.825	2.590	(-0.836,6.016)	0.134
Healthy women						
D	0.031	(-0.033,0.094)	0.339	0.246	(-0.329,0.820)	0.395
ND	0.035	(-0.037,0.106)	0.338	0.110	(-0.540,0.760)	0.736
**Ring fingertip average (ºC)**						
Women with FMS						
D	0.078	(-0.214,0.369)	0.592	3.064	(-0.269,6.397)	0.071
ND	0.033	(-0.269,0.334)	0.827	2.465	(-1.031,5.961)	0.162
Healthy women						
D	0.036	(-0.027,0.100)	0.257	0.215	(-0.362,0.791)	0.459
ND	0.043	(-0.027,0.112)	0.224	0.116	(-0.519,0.751)	0.716

Table S1. *Cont.*

	Serum free amino acids (pmoles/μL)					
	5-Methyl-histidine			Tyrosine		
**Variable**	**β**	**95 % CI**	***p*-value**	**β**	**95 % CI**	***p*-value**
**Dorsal sites of both hands**						
**Pinkie fingertip average (ºC)**						
Women with FMS						
D	0.070	(-0.225,0.365)	0.634	3.258	(-0.103,6.619)	0.057
ND	-0.004	(-0.304,0.296)	0.980	2.479	(-0.993,5.950)	0.157
Healthy women						
D	0.037	(-0.025,0.100)	0.239	0.201	(-0.368,0.771)	0.482
ND	0.043	(-0.025,0.111)	0.209	0.086	(-0.537,0.709)	0.783
**Dorsal centre average (ºC)**						
Women with FMS						
D	0.468	(-0.126,1.061)	0.119	6.032	(-0.976,13.040)	0.090
ND	0.248	(-0.291,0.787)	0.358	3.589	(-2.773,9.952)	0.261
Healthy women						
D	0.008	(-0.152,0.168)	0.918	0.667	(-0.764,2.099)	0.354
ND	-0.012	(-0.178,0.154)	0.886	0.665	(-0.821,2.151)	0.374

* Significance level $p<0.05$.

Note. Beta (β) represents the regression coefficient, adjusted for age, menopause status and body mass index. Abbreviations. FMS: Fibromyalgia syndrome; 95% CI: 95% confidence interval; pmoles/μL: picomoles of amino acid per microliter; ºC: celsius degree; D: dominant; ND: non-dominant.

Table S2. Correlations between serum free amino acids and temperature of the palmar site of both hands in women with Fibromyalgia and controls.

	**Serum free amino acids (pmoles/µL)**											
	**Phenylalanine**			**Methionine**			**Tryptophan**			**Threonine**		
**Variable**	**β**	**95 % CI**	***p*-value**	**β**	**95 % CI**	***p*-value**	**β**	**95 % CI**	***p*-value**	**β**	**95 % CI**	***p*-value**
**Palmar sites of both hands**												
**Thumb fingertip average (ºC)**												
Women with FMS												
D	1.617	(-1.867,5.100)	0.354	**1.995**	**(0.043,3.948)**	**0.045***	**9.714**	**(1.666,17.763)**	**0.019***	4.281	(-6.743,15.305)	0.437
ND	1.471	(-2.096,5.039)	0.409	1.686	(-0.343,3.714)	0.101	**9.116**	**(0.797,17.436)**	**0.033***	3.178	(-8.128,14.483)	0.573
Healthy women												
D	0.124	(-0.469,0.717)	0.676	0.004	(-0.168,0.176)	0.965	-0.187	(-1.217,0.843)	0.718	0.199	(-0.185,0.584)	0.303
ND	0.189	(-0.435,0.812)	0.547	-0.020	(-0.200,0.160)	0.827	-0.086	(-1.159,0.987)	0.873	0.087	(-0.319,0.492)	0.670
**Index fingertip average (ºC)**												
Women with FMS												
D	1.755	(-1.450,4.960)	0.275	**1.988**	**(0.200,3.777)**	**0.030***	**8.900**	**(1.455,16.345)**	**0.020***	5.697	(-4.403,15.798)	0.261
ND	1.428	(-1.824,4.681)	0.380	1.730	(-0.105,3.564)	0.064	**8.546**	**(0.978,16.115)**	**0.028***	4.533	(-5.727,14.792)	0.377
Healthy women												
D	0.084	(-0.438,0.606)	0.747	-0.020	(-0.171,0.132)	0.795	-0.282	(-1.187,0.622)	0.534	0.164	(-0.175,0.503)	0.338
ND	0.220	(-0.333,0.773)	0.428	-0.026	(-0.186,0.134)	0.744	-0.058	(-1.011,0.896)	0.904	0.056	(-0.304,0.416)	0.757
**Middle fingertip average (ºC)**												
Women with FMS												
D	1.526	(-1.704,4.756)	0.346	**1.978**	**(0.181,3.776)**	**0.032***	**8.798**	**(1.305,16.290)**	**0.023***	4.270	(-5.944,14.485)	0.403
ND	1.040	(-2.267,4.348)	0.529	1.523	(-0.354,3.400)	0.109	7.385	(-0.409,15.180)	0.063	2.697	(-7.754,13.149)	0.605
Healthy women												
D	0.061	(-0.463,0.586)	0.816	-0.030	(-0.182,0.123)	0.699	-0.232	(-1.141,0.678)	0.611	0.156	(-0.185,0.497)	0.362
ND	0.220	(-0.349,0.790)	0.441	-0.021	(-0.186,0.144)	0.799	0.037	(-0.945,1.019)	0.940	0.109	(-0.262,0.479)	0.559
**Ring fingertip average (ºC)**												
Women with FMS												
D	1.205	(-2.081,4.490)	0.463	1.587	(-0.275,3.448)	0.093	7.485	(-0.257,15.227)	0.058	4.622	(-5.708,14.952)	0.371
ND	1.075	(82.187,4.338)	0.509	1.708	(-0.127,3.542)	0.067	7.560	(-0.150,15.226)	0.053	2.610	(-7.706,12.926)	0.612
Healthy women												
D	0.035	(-0.476,0.546)	0.891	-0.036	(-0.184,0.112)	0.625	-0.138	(-1.026,0.749)	0.756	0.132	(-0.201,0.464)	0.431
ND	0.197	(-0.357,0.752)	0.479	-0.032	(-0.192,0.128)	0.692	-0.037	(-0.994,0.919)	0.938	0.107	(-0.254,0.468)	0.554

Table S2. *Cont.*

	Serum free amino acids (pmoles/µL)											
	Phenylalanine			Methionine			Tryptophan			Threonine		
**Variable**	**β**	**95 % CI**	***p*-value**	**β**	**95 % CI**	***p*-value**	**β**	**95 % CI**	***p*-value**	**β**	**95 % CI**	***p*-value**
**Palmar sites of both hands**												
**Pinkie fingertip average (ºC)**												
Women with FMS												
D	1.062	(-2.172,4.296)	0.511	1.610	(-0.216,3.435)	0.082	7.201	(-0.425,14.827)	0.064	5.461	(-4.647,15.569)	0.281
ND	1.048	(-2.107,4.203)	0.506	1.508	(-0.279,3.295)	0.096	7.290	(-0.126,14.705)	0.054	2.789	(-7.181,12.759)	0.575
Healthy women												
D	0.054	(-0.451,0.559)	0.831	-0.055	(-0.201,0.090)	0.449	-0.209	(-1.085,0.666)	0.634	0.187	(-0.140,0.513)	0.257
ND	0.251	(-0.274,0.776)	0.342	-0.029	(-0.181,0.123)	0.701	0.003	(-0.905,0.912)	0.994	0.097	(-0.245,0.440)	0.571
**Palm centre average (ºC)**												
Women with FMS												
D	0.094	(-7.321,7.508)	0.980	2.275	(-1.988,6.538)	0.287	12.630	(-5.079,30.339)	0.157	-1.420	(-24.805,21.965)	0.903
ND	0.258	(-6.764,7.279)	0.941	2.627	(-1.382,6.635)	0.193	11.905	(-4.870,28.681)	0.159	-3.452	(-25.575,18.672)	0.754
Healthy women												
D	0.078	(-1.415,1.571)	0.917	0.040	(-0.393,0.473)	0.853	0.230	(-2.364,2.823)	0.860	-0.161	(-1.138,0.815)	0.741
ND	0.241	(-1.283,1.766)	0.752	0.132	(-0.305,0.570)	0.546	0.881	(-1.725,3.488)	0.501	-0.337	(-1.323,0.648)	0.496
**Thenar eminence average (ºC)**												
Women with FMS												
D	-0.650	(-8.583,7.283)	0.869	2.704	(-1.844,7.251)	0.237	14.797	(-4.059,33.654)	0.121	2.078	(-22.947,27.103)	0.868
ND	-0.745	(-7.537,6.047)	0.826	2.558	(-1.321,6.437)	0.190	13.234	(-2.870,29.337)	0.105	-1.787	(-23.219,19.646)	0.867
Healthy women												
D	0.241	(-1.376,1.859)	0.766	-0.036	(-0.506,0.433)	0.877	-0.695	(-3.500,2.110)	0.621	0.182	(-0.876,1.240)	0.731
ND	0.165	(-1.449,1.780)	0.838	0.065	(-0.399,0.529)	0.781	-0.118	(-2.888,2.651)	0.932	0.340	(-0.704,1.383)	0.517
**Hypothenar eminence (ºC)**												
Women with FMS												
D	0.237	(-5.598,6.072)	0.935	2.054	(-1.286,5.394)	0.221	10.531	(-3.363,24.424)	0.133	3.827	(-14.541,22.194)	0.676
ND	-0.783	(-6.625,5.058)	0.788	1.937	(-1.417,5.291)	0.250	10.164	(-3.786,24.113)	0.149	-0.111	(-18.555,18.333)	0.990
Healthy women												
D	0.213	(-0.929,1.355)	0.710	-0.059	(-0.390,0.273)	0.723	-0.279	(-2.265,1.706)	0.779	0.175	(-0.572,0.922)	0.641
ND	0.436	(-0.740,1.611)	0.461	-0.086	(-0.425,0.252)	0.611	0.223	(-1.803,2.248)	0.826	0.085	(-0.681,0.851)	0.825

Table S2. *Cont.*

Variable	Serum free amino acids (pmoles/µL)											
	Lysine			Histidine			Alanine			Glycine		
	**β**	**95 % CI**	***p*-value**	**β**	**95 % CI**	***p*-value**	**β**	**95 % CI**	***p*-value**	**β**	**95 % CI**	***p*-value**
**Palmar sites of both hands**												
**Thumb fingertip average (ºC)**												
Women with FMS												
D	5.089	(-4.128,14.307)	0.271	2.014	(-0.766,4.793)	0.151	-4.555	(-14.080,4.970)	0.340	1.713	(-2.400,5.826)	0.405
ND	2.637	(-6.890,12.163)	0.579	1.667	(-1.199,4.534)	0.247	-6.955	(-16.546,2.637)	0.151	1.379	(-2.838,5.596)	0.513
Healthy women												
D	-0.315	(-0.815,0.185)	0.212	0.034	(-0.043,0.112)	0.379	1.136	(-0.353,2.624)	0.132	0.450	(-0.570,1.469)	0.380
ND	-0.467	(-0.989,0.054)	0.078	0.031	(-0.050,0.112)	0.442	1.013	(-0.549,2.574)	0.199	0.577	(-0.478,1.633)	0.278
**Index fingertip average (ºC)**												
Women with FMS												
D	5.598	(-2.862,14.059)	0.189	2.069	(-0.483,4.621)	0.109	-2.522	(-11.387,6.344)	0.569	1.625	(-2.173,5.424)	0.392
ND	4.270	(-4.353,12.893)	0.323	1.965	(-0.621,4.551)	0.132	-4.151	(-13.039,4.738)	0.351	1.056	(-2.800,4.911)	0.583
Healthy women												
D	-0.329	(-0.766,0.108)	0.137	0.039	(-0.029,0.107)	0.251	0.900	(-0.416,2.216)	0.176	0.298	(-0.602,1.198)	0.510
ND	-0.382	(-0.848,0.084)	0.106	0.032	(-0.040,0.104)	0.374	0.951	(-0.434,2.337)	0.174	0.694	(-0.235,1.624)	0.140
**Middle fingertip average (ºC)**												
Women with FMS												
D	5.263	(-3.255,13.781)	0.219	1.874	(-0.704,4.452)	0.150	-3.287	(-12.163,5.590)	0.459	1.128	(-2.704,4.961)	0.555
ND	2.708	(-6.086,11.501)	0.537	1.366	(-1.292,4.024)	0.305	-4.291	(-13.283,4.702)	0.341	0.365	(-3.551,4.281)	0.852
Healthy women												
D	-0.376	(-0.812,0.060)	0.090	0.037	(-0.032,0.105)	0.290	0.936	(-0.384,2.256)	0.161	0.477	(-0.421,1.376)	0.291
ND	-0.404	(-0.883,0.074)	0.096	0.040	(-0.034,0.114)	0.280	0.974	(-0.453,2.401)	0.177	0.730	(-0.226,1.686)	0.132
**Ring fingertip average (ºC)**												
Women with FMS												
D	3.948	(-4.753,12.649)	0.365	1.547	(-1.087,4.181)	0.242	-4.122	(-13.077,4.833)	0.358	0.387	(-3.509,4.283)	0.842
ND	3.449	(-5.201,12.099)	0.425	1.733	(-0.866,4.333)	0.185	-4.593	(-13.449,4.263)	0.301	0.528	(-3.334,4.391)	0.784
Healthy women												
D	-0.301	(-0.730,0.128)	0.165	0.034	(-0.033,0.101)	0.310	0.760	(-0.533,2.053)	0.244	0.432	(-0.444,1.308)	0.327
ND	-0.415	(-0.879,0.050)	0.079	0.041	(-0.030,0.113)	0.252	0.999	(-0.387,2.386)	0.154	0.755	(-0.173,1.683)	0.109

Table S2. *Cont.*

Variable	Serum free amino acids (pmoles/µL)											
	**Lysine**			**Histidine**			**Alanine**			**Glycine**		
	**β**	**95 % CI**	***p*-value**	**β**	**95 % CI**	***p*-value**	**β**	**95 % CI**	***p*-value**	**β**	**95 % CI**	***p*-value**
**Palmar sites of both hands**												
**Pinkie fingertip average (ºC)**												
Women with FMS												
D	3.583	(-4.984,12.149)	0.403	1.605	(-0.979,4.189)	0.217	-4.011	(-12.815,4.794)	0.363	0.451	(-3.378,4.280)	0.813
ND	2.529	(-5.866,10.923)	0.546	1.548	(-0.974,4.071)	0.222	-4.447	(-13.012,4.117)	0.300	0.823	(-2.907,4.552)	0.658
Healthy women												
D	-0.272	(-0.697,0.153)	0.205	0.031	(-0.035,0.098)	0.344	0.850	(-0.423,2.122)	0.186	0.465	(-0.399,1.329)	0.285
ND	-0.370	(-0.813,0.073)	0.100	0.028	(-0.040,0.097)	0.411	0.998	(-0.316,2.312)	0.134	0.720	(-0.162,1.602)	0.107
**Palm centre average (ºC)**												
Women with FMS												
D	5.641	(-13.984,25.265)	0.565	2.540	(-3.413,8.492)	0.394	-12.260	(-32.166,7.646)	0.220	3.220	(-5.457,11.896)	0.458
ND	3.163	(-15.473,21.799)	0.733	2.354	(-3.285,7.994)	0.404	-11.742	(-30.585,7.101)	0.215	4.223	(-3.940,12.387)	0.302
Healthy women												
D	-1.530	(-2.734,-0.325)	0.014	0.136	(-0.057,0.330)	0.163	1.526	(-2.277,5.329)	0.424	1.132	(-1.432,3.696)	0.380
ND	**-1.593**	**(-2.828,-0.358)**	**0.012***	0.099	(-0.097,0.296)	0.315	2.778	(-1.015,6.570)	0.148	1.596	(-0.970,4.162)	0.218
**Thenar eminence average (ºC)**												
Women with FMS												
D	5.601	(-15.415,26.618)	0.593	3.307	(-3.035,9.649)	0.298	-16.780	(-37.821,4.261)	0.115	4.824	(-4.400,14.047)	0.297
ND	3.100	(-14.937,21.137)	0.730	2.775	(-2.660,8.209)	0.308	-14.509	(-32.518,3.501)	0.111	4.725	(-3.141,12.590)	0.232
Healthy women												
D	**-1.805**	**(-3.096,-0.515)**	**0.007***	0.076	(-0.136,0.289)	0.474	0.533	(-3.611,4.677)	0.798	0.833	(-1.956,3.623)	0.552
ND	**-2.009**	**(-3.281,-0.737)**	**0.003***	0.076	(-0.133,0.284)	0.469	1.863	(-2.198,5.923)	0.362	1.137	(-1.599,3.874)	0.408
**Hypothenar eminence (ºC)**												
Women with FMS												
D	4.049	(-11.407,19.504)	0.599	2.788	(-1.855,7.432)	0.232	-10.729	(-26.324,4.865)	0.172	2.513	(-4.316,9.342)	0.461
ND	1.067	(-14.469,16.604)	0.890	2.887	(-1.759,7.534)	0.216	-9.078	(-24.811,6.655)	0.250	3.339	(-3.468,10.146)	0.327
Healthy women												
D	**-1.133**	**(-2.059,-0.207)**	**0.017***	0.047	(-0.104,0.197)	0.535	1.592	(-1.305,4.488)	0.275	0.821	(-1.144,2.786)	0.406
ND	**-1.214**	**(-2.172,-0.256)**	**0.014***	0.074	(-0.078,0.226)	0.335	2.034	(-0.909,4.977)	0.171	1.219	(-0.768,3.207)	0.224

Table S2. *Cont.*

	Serum free amino acids (pmoles/μL)											
	**Serine**			**Glutamine**			**Arginine**			**Taurine**		
**Variable**	**β**	**95 % CI**	***p*-value**	**β**	**95 % CI**	***p*-value**	**β**	**95 % CI**	***p*-value**	**β**	**95 % CI**	***p*-value**
**Palmar sites of both hands**												
**Thumb fingertip average (ºC)**												
Women with FMS												
D	0.601	(-7.175,8.377)	0.877	2.681	(-3.082,8.444)	0.353	0.680	(-2.318,3.678)	0.649	-1.389	(-5.133,2.355)	0.458
ND	-0.848	(-8.792,7.096)	0.830	2.251	(-3.659,8.160)	0.446	0.263	(-2.808,3.333)	0.863	-1.717	(-5.530,2.096)	0.368
Healthy women												
D	-0.376	(-1.068,0.315)	0.280	0.165	(-0.273,0.602)	0.454	-0.362	(-0.770,0.047)	0.082	-0.075	(-1.125,0.976)	0.887
ND	-0.564	(-1.276,0.148)	0.118	0.103	(-0.368,0.574)	0.663	-0.401	(-0.827,0.024)	0.064	-0.018	(-1.122,1.085)	0.973
**Index fingertip average (ºC)**												
Women with FMS												
D	1.348	(-5.826,8.522)	0.706	1.410	(-3.954,6.773)	0.598	1.135	(-1.618,3.889)	0.410	-1.401	(-4.855,2.054)	0.417
ND	0.425	(-6.829,7.679)	0.906	0.658	(-4.772,6.088)	0.808	0.488	(-2.312,3.288)	0.726	-1.835	(-5.303,1.632)	0.291
Healthy women												
D	-0.557	(-1.154,0.039)	0.066	0.047	(-0.339,0.434)	0.806	-0.249	(-0.613,0.115)	0.176	0.288	(-0.634,1.210)	0.533
ND	**-0.650**	**(-1.272,-0.027)**	**0.041***	0.089	(-0.329,0.508)	0.671	-0.219	(-0.605,0.167)	0.260	-0.033	(-1.013,0.948)	0.947
**Middle fingertip average (ºC)**												
Women with FMS												
D	1.544	(-5.655,8.743)	0.667	1.385	(-4.001,6.772)	0.606	0.799	(-1.978,3.576)	0.564	-1.151	(-4.629,2.327)	0.507
ND	0.129	(-7.214,7.473)	0.972	1.172	(-4.315,6.659)	0.668	0.244	(-2.593,3.081)	0.863	-2.215	(-5.703,1.273)	0.207
Healthy women												
D	-0.565	(-1.163,0.033)	0.064	0.047	(-0.342,0.435)	0.810	-0.211	(-0.579,0.156)	0.254	0.150	(-0.778,1.079)	0.746
ND	**-0.656**	**(-1.298,-0.014)**	**0.046***	0.122	(-0.308,0.533)	0.572	-0.222	(-0.619,0.175)	0.268	0.082	(-0.928,1.091)	0.872
**Ring fingertip average (ºC)**												
Women with FMS												
D	0.774	(-6.529,8.076)	0.832	1.465	(-3.988,6.918)	0.590	0.276	(-2.546,3.099)	0.844	-1.694	(-5.193,1.806)	0.334
ND	0.942	(-6.298,8.183)	0.794	1.595	(-3.809,6.998)	0.554	0.205	(-2.595,3.005)	0.883	-1.447	(-4.928,2.035)	0.406
Healthy women												
D	-0.507	(-1.093,0.079)	0.089	0.066	(-0.312,0.444)	0.728	-0.224	(-0.581,0.133)	0.213	0.044	(-0.861,0.949)	0.922
ND	-0.611	(-1.238,0.016)	0.056	0.090	(-0.330,0.509)	0.670	-0.234	(-0.620,0.152)	0.230	0.034	(-0.949,1.017)	0.945

Table S2. *Cont.*

Variable	Serine β	Serine 95 % CI	Serine *p*-value	Glutamine β	Glutamine 95 % CI	Glutamine *p*-value	Arginine β	Arginine 95 % CI	Arginine *p*-value	Taurine β	Taurine 95 % CI	Taurine *p*-value
	Serum free amino acids (pmoles/µL)											
**Palmar sites of both hands**												
**Pinkie fingertip average (ºC)**												
Women with FMS												
D	0.708	(-6.471,7.887)	0.843	1.615	(-3.740,6.969)	0.546	0.323	(-2.451,3.097)	0.815	-2.027	(-5.448,1.393)	0.238
ND	0.459	(-6.548,7.467)	0.895	1.367	(-3.864,6.599)	0.600	-0.020	(-2.729,2.688)	0.988	-1.486	(-4.850,1.877)	0.377
Healthy women												
D	-0.475	(-1.055,0.106)	0.107	0.053	(-0.321,0.426)	0.779	-0.199	(-0.553,0.154)	0.263	-0.067	(-0.961,0.827)	0.881
ND	-0.560	(-1.157,0.037)	0.065	0.081	(-0.318,0.480)	0.686	-0.198	(-0.565,0.170)	0.286	-0.022	(-0.955,0.912)	0.963
**Palm centre average (ºC)**												
Women with FMS												
D	-8.529	(-24.679,7.620)	0.292	5.272	(-6.879,17.422)	0.386	0.534	(-5.793,6.861)	0.865	-6.247	(-13.929,1.436)	0.108
ND	-7.870	(-23.175,7.436)	0.305	4.281	(-7.255,15.817)	0.458	1.017	(-4.968,7.003)	0.733	-4.884	(-12.238,2.469)	0.187
Healthy women												
D	-1.280	(-3.003,0.444)	0.142	-0.169	(-1.274,0.935)	0.759	-0.624	(-1.668,0.420)	0.236	0.504	(-2.136,3.144)	0.703
ND	**-1.908**	**(-3.606,-0.209)**	**0.028***	0.145	(-1.005,1.295)	0.801	-0.395	(-1.460,0.671)	0.461	-0.255	(-2.945,2.435)	0.850
**Thenar eminence average (ºC)**												
Women with FMS												
D	-5.697	(-23.131,11.738)	0.513	3.215	(-9.874,16.304)	0.622	-0.453	(-7.226,6.320)	0.893	-3.049	(-11.489,5.391)	0.470
ND	-5.094	(-20.018,9.830)	0.494	0.169	(-11.075,11.413)	0.976	-0.149	(-5.950,5.653)	0.959	-2.565	(-9.795,4.664)	0.477
Healthy women												
D	-0.937	(-2.826,0.952)	0.324	0.133	(-1.065,1.330)	0.825	-0.508	(-1.646,0.630)	0.375	-0.403	(-3.266,2.460)	0.799
ND	-1.355	(-3.199,0.488)	0.146	0.320	(-0.894,1.535)	0.599	-0.502	(-1.627,0.622)	0.374	-0.745	(-3.586,2.095)	0.601
**Hypothenar eminence (ºC)**												
Women with FMS												
D	-2.752	(-15.612,10.107)	0.668	3.208	(-6.392,12.807)	0.503	-0.683	(-5.660,4.294)	0.783	-2.852	(-9.032,3.329)	0.357
ND	-3.318	(-16.907,9.554)	0.605	0.807	(-8.863,10.476)	0.867	-1.047	(-6.027,3.933)	0.673	-2.263	(-8.481,3.954)	0.466
Healthy women												
D	-0.767	(-2.098,0.563)	0.253	0.001	(-0.846,0.847)	0.999	-0.357	(-1.161,0.448)	0.378	-0.723	(-2.738,1.291)	0.475
ND	-1.200	(-2.536,0.137)	0.077	0.123	(-0.767,1.014)	0.783	-0.195	(-1.022,0.633)	0.639	-0.805	(-2.877,1.267)	0.439

Table S2. *Cont.*

	Serum free amino acids (pmoles/µL)											
	**Glutamic acid**			**Asparagine**			**Aspartic acid**			**Ornithine**		
**Variable**	**β**	**95 % CI**	***p*-value**	**β**	**95 % CI**	***p*-value**	**β**	**95 % CI**	***p*-value**	**β**	**95 % CI**	***p*-value**
**Palmar sites of both hands**												
**Thumb fingertip average (ºC)**												
Women with FMS												
D	**4.018**	**(0.366,7.669)**	**0.032***	1.290	(-1.434,4.014)	0.344	0.200	(-1.513,1.913)	0.815	4.562	(-25.295,34.419)	0.759
ND	3.322	(-0.488,7.132)	0.086	0.896	(-1.904,3.696)	0.522	0.055	(-1.697,1.807)	0.950	9.090	(-21.317,39.496)	0.549
Healthy women												
D	0.124	(-0.139,0.387)	0.349	0.177	(-0.019,0.372)	0.075	0.060	(-0.200,0.320)	0.645	1.549	(-4.046,7.145)	0.581
ND	0.153	(-0.121,0.428)	0.268	0.153	(-0.056,0.362)	0.147	0.104	(-0.165,0.373)	0.442	2.290	(-3.564,8.143)	0.436
**Index fingertip average (ºC)**												
Women with FMS												
D	**4.321**	**(1.023,7.620)**	**0.012***	1.602	(-0.891,4.095)	0.201	0.109	(-1.474,1.693)	0.890	9.842	(-17.597,37.281)	0.473
ND	**3.798**	**(0.398,7.199)**	**0.029***	1.414	(-1.115,3.944)	0.265	0.126	(-1.473,1.724)	0.874	12.831	(-142748,40.410)	0.353
Healthy women												
D	0.116	(-0.115,0.347)	0.318	**0.172**	**(0.001,0.343)**	**0.049***	0.097	(-0.131,0.325)	0.397	1.632	(-3.287,6.551)	0.509
ND	0.124	(-0.120,0.369)	0.312	0.168	(-0.016,0.352)	0.072	0.072	(-0.167,0.312)	0.548	2.225	(-2.971,7.421)	0.394
**Middle fingertip average (ºC)**												
Women with FMS												
D	**4.051**	**(0.701,7.400)**	**0.019***	1.656	(-0.844,4.156)	0.188	0.140	(-1.450,1.730)	0.860	7.772	(-19.847,35.392)	0.573
ND	3.007	(-0.518,6.533)	0.092	0.982	(-1.599,3.564)	0.446	-0.001	(-1.619,1.617)	0.999	9.194	(-18.874,37.261)	0.512
Healthy women												
D	0.121	(-0.111,0.353)	0.302	**0.175**	**(0.004,0.347)**	**0.045***	0.092	(-0.137,0.320)	0.425	2.151	(-2.775,7.077)	0.385
ND	0.112	(-0.141,0.364)	0.379	0.147	(-0.043,0.338)	0.127	0.075	(-0.172,0.322)	0.546	2.393	(-2.953,7.739)	0.374
**Ring fingertip average (ºC)**												
Women with FMS												
D	3.067	(-0.434,6.569)	0.084	1.031	(-1.535,3.598)	0.421	0.074	(-1.536,1.684)	0.926	6.421	(-21.584,34.426)	0.646
ND	3.102	(-0.366,6.569)	0.078	1.102	(-1.440,3.644)	0.386	0.140	(-1.457,1.736)	0.860	5.746	(-22.043,33.535)	0.678
Healthy women												
D	0.135	(-0.090,0.360)	0.235	0.153	(-0.015,0.321)	0.074	0.071	(-0.152,0.294)	0.527	2.247	(-2.547,7.041)	0.351
ND	0.139	(-0.106,0.383)	0.260	0.143	(-0.043,0.328)	0.129	0.076	(-0.164,0.316)	0.529	2.701	(-2.491,7.893)	0.302

Table S2. *Cont.*

	Serum free amino acids (pmoles/µL)											
	**Glutamic acid**			**Asparagine**			**Aspartic acid**			**Ornithine**		
**Variable**	**β**	**95 % CI**	***p*-value**	**β**	**95 % CI**	***p*-value**	**β**	**95 % CI**	***p*-value**	**β**	**95 % CI**	***p*-value**
**Palmar sites of both hands**												
**Pinkie fingertip average (ºC)**												
Women with FMS												
D	3.270	(-0.148,6.688)	0.060	0.968	(-1.557,3.493)	0.443	0.001	(-1.583,1.583)	1.000	6.848	(-20.668,34.365)	0.618
ND	2.929	(-0.431,6.288)	0.086	1.034	(-1.426,3.494)	0.401	0.265	(-1.278,1.807)	0.730	7.512	(-19.315,34.340)	0.575
Healthy women												
D	0.139	(-0.083,0.361)	0.216	**0.166**	**(0.001,0.331)**	**0.048***	0.073	(-0.148,0.293)	0.512	2.674	(-2.042,7.391)	0.261
ND	0.114	(-0.119,0.347)	0.333	0.144	(-0.032,0.320)	0.107	0.072	(-0.156,0.301)	0.528	2.432	(-2.505,7.369)	0.328
**Palm centre average (ºC)**												
Women with FMS												
D	5.848	(-2.084,13.781)	0.144	0.797	(-4.997,6.592)	0.782	-1.762	(-5.327,1.803)	0.324	-2.146	(-65.082,60.791)	0.945
ND	5.385	(-2.139,12.909)	0.156	1.322	(-4.155,6.798)	0.628	-1.183	(-4.580,2.215)	0.486	-4.958	(-64.545,54.628)	0.867
Healthy women												
D	0.234	(-0.430,0.898)	0.483	0.035	(-0.472,0.541)	0.891	0.357	(-0.290,1.004)	0.274	0.586	(-13.529,14.700)	0.934
ND	0.239	(-0.435,0.913)	0.481	0.091	(-0.427,0.610)	0.726	0.136	(-0.523,0.795)	0.680	1.653	(-12.693,16.000)	0.818
**Thenar eminence average (ºC)**												
Women with FMS												
D	7.171	(-1.246,15.587)	0.093	2.017	(-4.157,8.191)	0.513	-1.111	(-4.958,2.735)	0.563	9.313	(-57.987,76.612)	0.781
ND	5.901	(-1.328,13.129)	0.107	2.365	(-2.897,7.628)	0.369	-0.566	(-3.870,2.737)	0.731	3.906	(-53.774,61.586)	0.892
Healthy women												
D	0.177	(-0.544,0.899)	0.624	0.112	(-0.436,0.660)	0.684	0.479	(-0.218,1.176)	0.174	5.715	(-9.503,20.933)	0.455
ND	0.304	(-0.408,1.016)	0.396	0.043	(-0.506,0.592)	0.876	0.270	(-0.424,0.965)	0.438	4.612	(-10.526,19.749)	0.544
**Hypothenar eminence (ºC)**												
Women with FMS												
D	4.782	(-1.447,11.012)	0.129	1.918	(-2.605,6.441)	0.397	-0.629	(-3.463,2.205)	0.656	11.280	(-38.124,60.685)	0.647
ND	4.614	(-1.641,10.869)	0.144	2.002	(-2.526,6.531)	0.377	-0.392	(-3.236,2.452)	0.782	7.162	(-42.417,56.741)	0.772
Healthy women												
D	0.138	(-0.372,0.647)	0.590	0.112	(-0.274,0.499)	0.563	0.398	(-0.091,0.888)	0.108	5.210	(-5.504,15.924)	0.334
ND	0.225	(-0.295,0.746)	0.389	0.071	(-0.331,0.472)	0.726	0.262	(-0.244,0.768)	0.304	4.262	(-6.792,15.316)	0.443

Table S2. *Cont.*

	Serum free amino acids (pmoles/µL)											
	**Citrulline**			**Aminoadipic acid**			**Carnosine**			**γ-aminobutyric acid (GABA)**		
**Variable**	**β**	**95 % CI**	***p*-value**	**β**	**95 % CI**	***p*-value**	**β**	**95 % CI**	***p*-value**	**β**	**95 % CI**	***p*-value**
**Palmar sites of both hands**												
**Thumb fingertip average (ºC)**												
Women with FMS												
D	0.394	(-1.036,1.824)	0.580	0.034	(-0.079,0.146)	0.550	2.197	(-10.324,12.087)	0.733	2.810	(-5.239,10.858)	0.485
ND	0.409	(-1.052,1.870)	0.575	0.019	(-0.097,0.134)	0.746	-0.099	(-13.314,13.116)	0.988	1.634	(-6.624,9.893)	0.691
Healthy women												
D	0.139	(-0.264,0.541)	0.492	-0.013	(-0.041,0.015)	0.343	1.766	(-0.746,4.278)	0.164	-1.455	(-4.738,1.827)	0.378
ND	0.098	(-0.321,0.516)	0.642	-0.011	(-0.041,0.019)	0.466	0.651	(-2.025,3.326)	0.628	-1.003	(-4.494,2.487)	0.567
**Index fingertip average (ºC)**												
Women with FMS												
D	0.732	(-0.573,2.038)	0.264	0.046	(-0.058,0.149)	0.378	5.543	(-6.274,17.360)	0.349	4.348	(-3.004,11.700)	0.239
ND	0.695	(-0.625,2.015)	0.294	0.040	(-0.064,0.145)	0.439	4.115	(-7.876,16.106)	0.492	2.666	(-4.840,10.171)	0.477
Healthy women												
D	0.142	(-0.212,0.495)	0.425	-0.015	(-0.039,0.010)	0.235	1.202	(-1.026,3.430)	0.284	-0.916	(-3.816,1.983)	0.529
ND	0.086	(-0.286,0.458)	0.645	-0.013	(-0.040,0.013)	0.318	0.503	(-1.876,2.882)	0.673	-0.739	(-3.8433,2.366)	0.635
**Middle fingertip average (ºC)**												
Women with FMS												
D	0.826	(-0.480,2.131)	0.208	0.039	(-0.065,0.143)	0.457	4.955	(-6.937,16.848)	0.405	3.799	(-3.615,11.212)	0.307
ND	0.462	(-0.886,1.809)	0.493	0.021	(-0.086,0.127)	0.696	1.609	(-10.589,13.808)	0.791	1.001	(-6.637,8.639)	0.792
Healthy women												
D	0.117	(-0.239,0.473)	0.512	-0.014	(-0.039,0.010)	0.246	0.995	(-1.251,3.241)	0.378	-1.121	(-4.029,1.786)	0.443
ND	0.059	(-0.324,0.442)	0.758	-0.017	(-0.044,0.010)	0.211	0.679	(-1.767,3.125)	0.580	-1.236	(-4.421,1.950)	0.440
**Ring fingertip average (ºC)**												
Women with FMS												
D	0.536	(-0.801,1.874)	0.422	0.025	(-0.081,0.131)	0.636	3.308	(-8.794,15.411)	0.584	2.212	(-5.362,9.786)	0.558
ND	0.621	(-0.701,1.944)	0.348	0.018	(-0.087,0.123)	0.734	1.904	(-10.129,13.938)	0.751	1.938	(-5.581,9.457)	0.605
Healthy women												
D	0.141	(-0.205,0.487)	0.416	-0.014	(-0.038,0.011)	0.263	1.147	(-1.035,3.328)	0.297	-0.877	(-3.715,1.961)	0.538
ND	0.086	(-0.287,0.458)	0.647	-0.013	(-0.040,0.013)	0.311	0.611	(-1.772,2.994)	0.609	-1.052	(-4.158,2.054)	0.500

Table S2. *Cont.*

	Serum free amino acids (pmoles/µL)											
	**Citrulline**			**Aminoadipic acid**			**Carnosine**			**γ-aminobutyric acid (GABA)**		
**Variable**	**β**	**95 % CI**	***p*-value**	**β**	**95 % CI**	***p*-value**	**β**	**95 % CI**	***p*-value**	**β**	**95 % CI**	***p*-value**
**Palmar sites of both hands**												
**Pinkie fingertip average (ºC)**												
Women with FMS												
D	0.750	(-0.554,2.053)	0.252	0.038	(-0.066,0.142)	0.463	4.992	(-6.843,16.827)	0.399	2.101	(-5.346,9.549)	0.572
ND	0.566	(-0.715,1.847)	0.377	0.016	(-0.085,0.118)	0.748	1.956	(-9.680,13.592)	0.736	1.888	(-5.384,9.159)	0.603
Healthy women												
D	0.121	(-0.222,0.463)	0.483	-0.011	(-0.035,0.013)	0.366	1.262	(-0.887,3.411)	0.244	-1.302	(-4.092,1.489)	0.354
ND	0.042	(-0.312,0.397)	0.812	-0.012	(-0.037,0.013)	0.334	0.320	(-1.948,2.587)	0.778	-0.768	(-3.723,2.187)	0.605
**Palm centre average (ºC)**												
Women with FMS												
D	0.150	(-2872,3.172)	0.920	-0.054	(-0.292,0.183)	0.645	2.807	(-24.408,30.023)	0.836	-0.075	(-17.126,16.976)	0.993
ND	0.560	(-2.297,3.416)	0.694	-0.045	(-0.270,0.180)	0.687	-0.504	(-26.292,25.284)	0.969	3.761	(-12.342,19.864)	0.639
Healthy women												
D	0.293	(-0.720,1.307)	0.564	-0.042	(-0.112,0.028)	0.237	3.755	(-2.598,10.108)	0.241	-0.213	(-8.530,8.105)	0.959
ND	0.201	(-0.820,1.221)	0.695	-0.051	(-0.123,0.021)	0.161	1.883	(-4.635,8.401)	0.565	-1.593	(-10.119,6.932)	0.709
**Thenar eminence average (ºC)**												
Women with FMS												
D	1.083	(-2.133,4.300)	0.500	-0.042	(-0.297,0.212)	0.739	6.846	(-22.216,35.909)	0.637	3.443	(-14.773,21.660)	0.704
ND	1.091	(-1.658,3.839)	0.427	-0.047	(-0.265,0.170)	0.664	1.791	(-23.163,26.745)	0.885	4.220	(-11.351,19.791)	0.587
Healthy women												
D	0.314	(-0.784,1.413)	0.568	-0.067	(-0.142,0.008)	0.079	5.249	(-1.576,12.073)	0.129	-0.663	(-9.676,8.350)	0.883
ND	0.328	(-0.750,1.406)	0.545	-0.082	(-0.156,-0.007)	0.032	1.869	(-5.031,8.769)	0.589	-1.781	(-10.801,7.240)	0.694
**Hypothenar eminence (ºC)**												
Women with FMS												
D	0.830	(-1.534,3.194)	0.482	-0.010	(-0.197,0.178)	0.918	5.428	(-15.933,26.789)	0.610	1.779	(-11.629,15.186)	0.790
ND	0.960	(-1.403,3.324)	0.416	-0.025	(-0.212,0.163)	0.790	4.387	(-17.039,25.814)	0.681	2.997	(-10.414,16.409)	0.654
Healthy women												
D	-0.066	(-0.845,0.712)	0.865	-0.025	(-0.079,0.029)	0.356	1.609	(-3.300,6.518)	0.514	0.725	(-5.642,7.091)	0.820
ND	-0.009	(-0.800,0.783)	0.983	-0.034	(-0.090,0.022)	0.228	0.535	(-4.525,5.596)	0.833	-1.151	(-7.754,5.452)	0.728

Table S2. *Cont.*

	Serum free amino acids (pmoles/µL)											
	**Isoleucine**			**Leucine**			**Valine**			**3-Methyl-histidine**		
**Variable**	**β**	**95 % CI**	***p*-value**	**β**	**95 % CI**	***p*-value**	**β**	**95 % CI**	***p*-value**	**β**	**95 % CI**	***p*-value**
**Palmar sites of both hands**												
**Thumb fingertip average (ºC)**												
Women with FMS												
D	1.802	(-2.579,6.183)	0.411	6.562	(-4.416,17.540)	0.234	6.525	(-10.418,23.468)	0.441	11.397	(-0.487,23.281)	0.060
ND	0.709	(-3.800,5.219)	0.752	5.515	(-5.769,16.799)	0.329	5.156	(-12.209,22.522)	0.552	9.131	(-3.230,21.492)	0.143
Healthy women												
D	0.384	(-0.302,1.069)	0.267	0.420	(-1.439,2.278)	0.652	0.036	(-0.434,0.505)	0.879	0.091	(-0.965,1.148)	0.863
ND	0.342	(-0.381,1.065)	0.348	0.613	(-1.311,2.537)	0.526	0.023	(-0.465,0.511)	0.926	0.456	(-0.633,1.546)	0.405
**Index fingertip average (ºC)**												
Women with FMS												
D	2.086	(-1.942,6.114)	0.301	7.176	(-2.893,17.244)	0.158	5.997	(-9.659,21.652)	0.443	**10.994**	**(0.061,21.927)**	**0.049***
ND	1.503	(-2.591,5.596)	0.462	6.238	(-3.994,16.471)	0.225	5.515	(-10.310,21.339)	0.485	8.960	(-2.274,20.194)	0.115
Healthy women												
D	0.363	(-0.239,0.966)	0.232	0.013	(-1.627,1.652)	0.988	-0.051	(-0.463,0.362)	0.807	0.104	(-0.825,1.034)	0.823
ND	0.278	(-0.366,0.921)	0.391	0.274	(-1.440,1.989)	0.750	-0.090	(-0.524,0.343)	0.678	0.452	(-0.515,1.419)	0.353
**Middle fingertip average (ºC)**												
Women with FMS												
D	1.757	(-2.303,5.818)	0.387	6.713	(-3.431,16.857)	0.189	3.706	(-12.086,19.498)	0.638	**11.729**	**(0.825,22.633)**	**0.036***
ND	0.517	(-3.651,4.686)	0.803	4.724	(-5.719,15.167)	0.366	2.318	(-13.781,18.417)	0.773	7.961	(-3.494,19.416)	0.168
Healthy women												
D	0.378	(-0.227,0.982)	0.216	0.180	(-1.466,1.826)	0.827	-0.057	(-0.471,0.358)	0.785	0.169	(-0.764,1.102)	0.718
ND	0.288	(-0.374,0.950)	0.387	-0.024	(-1.791,1.743)	0.978	-0.088	(-0.534,0.358)	0.693	0.488	(-0.507,1.482)	0.330
**Ring fingertip average (ºC)**												
Women with FMS												
D	1.035	(-3.102,5.173)	0.616	6.067	(-4.251,16.386)	0.242	2.348	(-13.670,18.366)	0.769	9.432	(-1.849,20.712)	0.099
ND	0.090	(-4.027,4.207)	0.965	4.325	(-5.996,14.646)	0.402	0.100	(-15.804,16.004)	0.990	9.516	(-1.657,20.690)	0.093
Healthy women												
D	0.284	(-0.308,0.877)	0.340	0.025	(-1.579,1.629)	0.975	-0.077	(-0.481,0.327)	0.704	0.241	(-0.667,1.149)	0.597
ND	0.280	(-0.365,0.925)	0.388	0.141	(-1.579,1.861)	0.870	-0.070	(-0.505,0.364)	0.747	0.520	(-0.447,1.487)	0.286

Table S2. *Cont.*

Variable	Serum free amino acids (pmoles/µL)											
	Isoleucine			Leucine			Valine			3-Methyl-histidine		
	β	95 % CI	*p*-value	β	95 % CI	*p*-value	β	95 % CI	*p*-value	β	95 % CI	*p*-value
**Palmar sites of both hands**												
**Pinkie fingertip average (ºC)**												
Women with FMS												
D	0.945	(-3.125,5.014)	0.641	5.312	(-4.868,15.492)	0.298	1.984	(-13.767,17.734)	0.800	10.186	(-0.820,21.192)	0.069
ND	0.448	(-3.531,4.427)	0.821	4.320	(-5.655,14.296)	0.387	0.736	(-14.643,16.116)	0.923	8.104	(-2.791,19.000)	0.141
Healthy women												
D	0.302	(-0.282,0.887)	0.304	-0.104	(-1.688,1.480)	0.896	-0.078	(-0.476,0.321)	0.698	0.250	(-0.646,1.146)	0.578
ND	0.329	(-0.281,0.940)	0.284	0.227	(-1.406,1.860)	0.782	-0.063	(-0.476,0.350)	0.762	0.489	(-0.430,1.407)	0.291
**Palm centre average (ºC)**												
Women with FMS												
D	-0.604	(-9.906,8.698)	0.896	2.215	(-21.308,25.737)	0.850	-2.434	(-38.369,33.500)	0.892	16.555	(-9.075,42.185)	0.199
ND	-2.160	(-10.944,6.624)	0.622	1.643	(-20.638,23.924)	0.882	-2.938	(-36.965,31.089)	0.862	17.874	(-6.244,41.992)	0.142
Healthy women												
D	0.506	(-1.233,2.245)	0.562	2.861	(-1.757,7.478)	0.219	0.057	(-1.123,1.238)	0.923	-0.603	(-3.256,2.050)	0.650
ND	0.122	(-1.655,1.900)	0.891	3.699	(-0.900,8.299)	0.113	-0.050	8-1.241,1.141)	0.933	0.390	(-2.282,3.062)	0.771
**Thenar eminence average (ºC)**												
Women with FMS												
D	-1.019	(-10.972,8.933)	0.837	0.048	(-25.139,25.236)	0.997	-7.460	(-45.856,30.936)	0.697	16.338	(-11.182,43.857)	0.237
ND	-2.796	(-11.277,5.686)	0.509	-0.916	(-22.486,20.653)	0.932	-5.356	(-38.259,27.546)	0.744	16.491	(-6.912,39.894)	0.162
Healthy women												
D	0.460	(-1.427,2.347)	0.627	1.321	(-3.743,6.385)	0.603	0.415	(-0.860,1.689)	0.517	-0.046	(-2.926,2.835)	0.975
ND	0.062	(-1.819,1.943)	0.948	2.389	(-2.551,7.330)	0.337	0.249	(-1.009,1.507)	0.693	0.252	(-2.577,3.081)	0.859
**Hypothenar eminence (ºC)**												
Women with FMS												
D	-0.946	(-8.262,6.370)	0.795	2.210	(-16.298,20.718)	0.811	-0.057	(-28.345,28.231)	0.997	11.080	(-9.211,31.370)	0.276
ND	-3.069	(-10.340,4.202)	0.399	-1.244	(-19.797,17.308)	0.893	-6.358	(-34.629,21.912)	0.652	11.864	(-8.421,32.150)	0.244
Healthy women												
D	0.396	(-0.936,1.727)	0.554	0.884	(-2.695,4.463)	0.622	0.012	(-0.892,0.916)	0.978	-0.246	(-2.281,1.788)	0.809
ND	0.249	(-1.125,1.624)	0.718	0.894	(-2.744,4.532)	0.624	0.026	(-0.896,0.948)	0.954	0.647	(-1.416,2.710)	0.532

Table S2. *Cont.*

	Serum free amino acids (pmoles/µL)					
	5-Methyl-histidine			Tyrosine		
**Variable**	**β**	**95 % CI**	***p*-value**	**β**	**95 % CI**	***p*-value**
**Palmar sites of both hands**						
**Thumb fingertip average (ºC)**						
Women with FMS						
D	0.044	(-0.281,0.368)	0.788	2.837	(-0.916,6.589)	0.134
ND	-0.006	(-0.339,0.326)	0.969	2.338	(-1.535,6.212)	0.230
Healthy women						
D	0.041	(-0.032,0.114)	0.263	0.300	(-0.362,0.963)	0.367
ND	0.061	(-0.015,0.137)	0.114	0.148	(-0.552,0.848)	0.673
**Index fingertip average (ºC)**						
Women with FMS						
D	0.129	(-0.169,0.426)	0.386	**3.461**	**(0.070,6.852)**	**0.046***
ND	0.089	(-0.213,0.391)	0.556	3.011	(-0.459,6.481)	0.087
Healthy women						
D	0.041	(-0.023,0.105)	0.208	0.153	(-0.433,0.739)	0.603
ND	0.042	(-0.027,0.110)	0.228	0.023	(-0.600,0.646)	0.941
**Middle fingertip average (ºC)**						
Women with FMS						
D	0.070	(-0.231,0.370)	0.643	**3.485**	**(0.081,6.889)**	**0.045***
ND	0.018	(-0.289,0.324)	0.908	2.561	(-0.991,6.113)	0.153
Healthy women						
D	0.038	(-0.027,0.102)	0.245	0.125	(-0.464,0.714)	0.672
ND	0.042	(-0.028,0.112)	0.235	0.018	(-0.623,0.660)	0.955
**Ring fingertip average (ºC)**						
Women with FMS						
D	0.035	(-0.270,0.340)	0.816	2.802	(-0.712,6.317)	0.115
ND	0.014	(-0.289,0.317)	0.926	2.590	(-0.910,6.091)	0.143
Healthy women						
D	0.042	(-0.021,0.104)	0.184	0.112	(-0.462,0.686)	0.697
ND	0.051	(-0.017,0.119)	0.136	0.027	(-0.598,0.651)	0.932

Table S2. *Cont.*

Variable	Serum free amino acids (pmoles/µL)					
	5-Methyl-histidine			Tyrosine		
	**β**	**95 % CI**	***p*-value**	**β**	**95 % CI**	***p*-value**
**Palmar sites of both hands**						
**Pinkie fingertip average (ºC)**						
Women with FMS						
D	0.049	(-0.250,0.349)	0.741	3.033	(-0.398,6.464)	0.082
ND	0.005	(-0.288,0.298)	0.972	2.684	(-0.687,6.055)	0.115
Healthy women						
D	0.042	(-0.019,0.104)	0.174	0.131	(-0.436,0.698)	0.645
ND	0.045	(-0.020,0.109)	0.173	-0.009	(-0.603,0.584)	0.975
**Palm centre average (ºC)**						
Women with FMS						
D	0.092	(-0.592,0.776)	0.787	3.141	(-4.926,11.207)	0.436
ND	0.006	(-0.642,0.655)	0.984	2.787	(-4.860,10.4339	0.466
Healthy women						
D	0.107	(-0.076,0.290)	0.247	0.287	(-1.390,1.964)	0.733
ND	0.075	(-0.113,0.264)	0.427	0.372	(-1.335,2.078)	0.664
**Thenar eminence average (ºC)**						
Women with FMS						
D	-0.004	(-0.736,0.729)	0.992	3.827	(-4.787,12.441)	0.375
ND	-0.122	(-0.749,0.504)	0.695	1.570	(-5.864,9.005)	0.672
Healthy women						
D	0.145	(-0.052,0.342)	0.146	0.217	(-1.601,2.036)	0.812
ND	0.083	(-0.117,0.282)	0.410	0.188	(-1.620,1.997)	0.835
**Hypothenar eminence (ºC)**						
Women with FMS						
D	-0.060	(-0.599,0.478)	0.822	3.271	(-3.041,9.582)	0.301
ND	-0.131	(-0.669,0.408)	0.626	1.729	(-4.658,8.115)	0.587
Healthy women						
D	0.124	(-0.014,0.262)	0.076	-0.041	(-1.327,1.244)	0.949
ND	0.103	(-0.042,0.247)	0.160	-0.299	(-1.621,1.022)	0.651

* Significance level $p<0.05$.

Note. Beta (β) represents the regression coefficient, adjusted for age, menopause status and body mass index. Abbreviations. FMS: Fibromyalgia syndrome; 95% CI: 95% confidence interval; pmoles/µL: picomoles of amino acid per microliter; ºC: celsius degree; D: dominant; ND: non-dominant.

Table S3. Associations between serum free amino acids and central temperature in women with Fibromyalgia and controls.

	Serum free amino acids (pmoles/µL)											
	**Phenylalanine**			**Methionine**			**Tryptophan**			**Threonine**		
**Variable**	**β**	**95 % CI**	***p*-value**	**β**	**95 % CI**	***p*-value**	**β**	**95 % CI**	***p*-value**	**β**	**95 % CI**	***p*-value**
**Tympanic temperature (ºC)**												
Women with FMS	3.459	(-11.809,18.727)	0.649	1.349	(-7.474,10.173)	0.759	5.922	(-32.435,44.279)	0.757	16.790	(-31.266,15.867)	0.484
Healthy women	2.202	(-0.526,4.930)	0.111	-0.102	(-0.904,0.701)	0.801	0.148	(-4.643,4.938)	0.951	1.377	(-0.396,3.150)	0.125
**Axillary temperature (ºC)**												
Women with FMS	4.530	(-6.814,15.874)	0.422	4.261	(-2.138,10.660)	0.184	9.575	(-19.092,38.242)	0.501	9.647	(-26.491,45.785)	0.590
Healthy women	1.799	(-1.041,4.639)	0.209	0.120	(-0.644,0.884)	0.753	**5.340**	**(0.903,9.778)**	**0.019***	0.526	(-1.014,2.065)	0.495

	Serum free amino acids (pmoles/µL)											
	**Lysine**			**Histidine**			**Alanine**			**Glycine**		
**Variable**	**β**	**95 % CI**	***p*-value**	**β**	**95 % CI**	***p*-value**	**β**	**95 % CI**	***p*-value**	**β**	**95 % CI**	***p*-value**
**Tympanic temperature (ºC)**												
Women with FMS	15.435	(-25.495,56.365)	0.450	5.627	(-6.705,17.958)	0.362	-0.287	(-44.523,43.949)	0.990	-3.650	(-22.418,15.118)	0.696
Healthy women	-0.548	(-2.939,1.844)	0.648	-0.156	(-0.516,0.204)	0.390	-0.503	(-7.581,6.575)	0.887	-0.252	(-5.015,4.512)	0.916
**Axillary temperature (ºC)**												
Women with FMS	0.973	(-30.797,32.743)	0.951	1.697	(-7.899,11.294)	0.721	-3.017	(-34.756,28.722)	0.848	-3.725	(-17.230,9.779)	0.578
Healthy women	-1.201	(-3.470,1.067)	0.292	-0.128	(-0.500,0.243)	0.491	-1.174	(-8.202,5.853)	0.738	1.786	(-2.752,6.325)	0.432

	Serum free amino acids (pmoles/µL)											
	**Serine**			**Glutamine**			**Arginine**			**Taurine**		
**Variable**	**β**	**95 % CI**	***p*-value**	**β**	**95 % CI**	***p*-value**	**β**	**95 % CI**	***p*-value**	**β**	**95 % CI**	***p*-value**
**Tympanic temperature (ºC)**												
Women with FMS	-1.732	(-37.488,34.024)	0.922	4.890	(-20.860,30.639)	0.703	-3.561	(-17.038,9.917)	0.596	0.086	(-16.923,17.095)	0.992
Healthy women	-0.718	(-3.965,2.528)	0.659	-1.511	(-3.577,0.554)	0.148	-0.216	(-2.175,1.743)	0.826	-3.903	(-8.712,0.907)	0.110
**Axillary temperature (ºC)**												
Women with FMS	1.664	(-25.228,28.555)	0.900	12.871	(-6.509,32.251)	0.185	8.286	(-1.737,18.309)	0.102	4.324	(-7.723,16.371)	0.470
Healthy women	-1.201	(-4.207,1.805)	0.425	0.505	(-1.561,2.570)	0.625	-0.254	(-2.197,1.689)	0.793	1.196	(-3.625,6.018)	0.620

Table S3. *Cont.*

	Serum free amino acids (pmoles/μL)											
	**Glutamic acid**			**Asparagine**			**Aspartic acid**			**Ornithine**		
**Variable**	**β**	**95 % CI**	***p*-value**	**β**	**95 % CI**	***p*-value**	**β**	**95 % CI**	***p*-value**	**β**	**95 % CI**	***p*-value**
**Tympanic temperature (ºC)**												
Women with FMS	7.736	(-8.721,24.192)	0.348	-1.813	(-14.233,10.607)	0.982	0.700	(-7.005,8.404)	0.855	-17.024	(-149.339,115.290)	0.796
Healthy women	1.184	(-0.013,2.381)	0.052	0.157	(-0.793,1.107)	0.742	0.156	(-1.051,1.364)	0.796	-10.614	(-36.729,15.500)	0.419
**Axillary temperature (ºC)**												
Women with FMS	0.635	(-11.439,12.709)	0.915	-3.131	(-12.357,6.094)	0.494	-0.634	(-6.242,4.973)	0.819	23.890	(-74.459,122.238)	0.624
Healthy women	-0.004	(-1.220,1.212)	0.995	-0.110	(-1.019,0.799)	0.808	-0.667	(-1.837,0.503)	0.257	10.487	(-14.568,35.541)	0.404

	Serum free amino acids (pmoles/μL)											
	**Citrulline**			**Aminoadipic acid**			**Carnosine**			**γ-aminobutyric acid (GABA)**		
**Variable**	**β**	**95 % CI**	***p*-value**	**β**	**95 % CI**	***p*-value**	**β**	**95 % CI**	***p*-value**	**β**	**95 % CI**	***p*-value**
**Tympanic temperature (ºC)**												
Women with FMS	-2.252	(-8.595,4.091)	0.477	0.147	(-0.347,0.642)	0.550	39.625	(-17.395,96.645)	0.168	4.496	(-32.528,41.521)	0.807
Healthy women	1.486	(-0.341,3.312)	0.109	-0.056	(-0.189,0.077)	0.404	1.239	(-10.724,13.203)	0.836	-10.832	(-26.176,4.512)	0.163
**Axillary temperature (ºC)**												
Women with FMS	-1.453	(-6.160,3.254)	0.534	0.036	(-0.326,0.398)	0.841	2.059	(-42.610,46.728)	0.926	-15.652	(-41.459,10.154)	0.225
Healthy women	**2.038**	**(0.343,3.733)**	**0.020***	**-0.138**	**(-0.266,-0.009)**	**0.036***	-8.106	(-19.893,3.682)	0.173	1.261	(-13.603,16.125)	0.865

	Serum free amino acids (pmoles/μL)											
	**Isoleucine**			**Leucine**			**Valine**			**3-Methyl-histidine**		
**Variable**	**β**	**95 % CI**	***p*-value**	**β**	**95 % CI**	***p*-value**	**β**	**95 % CI**	***p*-value**	**β**	**95 % CI**	***p*-value**
**Tympanic temperature (ºC)**												
Women with FMS	12.420	(-5.706,30.546)	0.174	**48.045**	**(3.503,92.587)**	**0.035***	-14.457	(-86.588,57.674)	0.688	-12.055	(-67.318,43.207)	0.662
Healthy women	-2.363	(-5.552,0.826)	0.143	0.910	(-7.706,9.526)	0.833	1.709	(-0.420,3.838)	0.113	1.522	(-3.356,6.400)	0.534
**Axillary temperature (ºC)**												
Women with FMS	7.537	(-6.821,21.894)	0.293	**35.871**	**(1.847,69.895)**	**0.039***	27.259	(-26.320,80.837)	0.307	0.873	(-41.222,42.968)	0.967
Healthy women	-2.833	(-6.100,0.434)	0.088	-3.786	(-12.334,4.763)	0.377	-0.902	(-3.030,1.227)	0.398	2.415	(-2.357,7.188)	0.313

Table S3. *Cont.*

	Serum free amino acids (pmoles/µL)					
	5-Methyl-histidine			Tyrosine		
**Variable**	**β**	**95 % CI**	***p*-value**	**β**	**95 % CI**	***p*-value**
**Tympanic temperature (ºC)**						
Women with FMS	0.330	(-1.091,1.751)	0.642	0.677	(-15.738,17.093)	0.934
Healthy women	-0.119	(-0.465,0.227)	0.494	-0.677	(-3.801,2.447)	0.666
**Axillary temperature (ºC)**						
Women with FMS	0.480	(-0.538,1.498)	0.344	4.662	(-7.649,16.973)	0.446
Healthy women	-0.117	(-0.453,0.220)	0.488	-0.652	(-3.694,2.389)	0.668

* Significance level $p<0.05$.

Note. Beta (β) represents the regression coefficient, adjusted for age, menopause status and body mass index. Abbreviations. FMS: Fibromyalgia syndrome; 95% CI: 95% confidence interval; pmoles/µL: picomoles of amino acid per microliter; ºC: celsius degree.
